# Supplementary material for: Unified failure model for landslides, rockbursts, glaciers, and volcanoes
Source: Commun Earth Environ. 2025 May 20;6(1):390. doi: 10.1038/s43247-025-02369-z (PMC12092298; doi:10.1038/s43247-025-02369-z)
Supplement: Supplementary file 2 — Supplementary Information [file 43247_2025_2369_MOESM2_ESM.pdf]

Supplementary Information for:

Unified failure model for landslides, rockbursts, glaciers, and volcanoes

Qinghua Lei[1*], Didier Sornette[2]

[1]*Department of Earth Sciences, Uppsala University; Uppsala, Sweden*

[2]*Institute of Risk Analysis, Prediction and Management, Academy for Advanced Interdisciplinary Studies, Southern University of Science and Technology; Shenzhen, China*

This PDF file includes:

Supplementary Notes 1-7

Supplementary Figures 1-15

Supplementary Tables 1-16

[*] Corresponding author. Email: qinghua.lei@geo.uu.se

Supplementary Note 1. Derivation of the power law singularity (PLS) model

The response of a heterogeneous material prior to a catastrophic failure is generically governed by the following nonlinear dynamic equation[1,2]:

$$\ddot{\Omega} = \eta \dot{\Omega}^{\alpha}\text{, with } \alpha > 1, \tag{S1}$$

where Ω is an observable quantity, η is a constant, and α defines the degree of nonlinearity. The condition $\alpha > 1$ ensures the presence of positive feedbacks[3,4], where the instantaneous growth rate of $\dot{\Omega}$ defined as $\mathrm{d}(\ln \dot{\Omega}) / \mathrm{d}t$ increases super-linearly as a function of $\dot{\Omega}$. This corresponds to a super-exponential dynamic ending with a finite-time singularity at which a catastrophic failure occurs. This is seen by integrating equation (S1), which yields:

$$\dot{\Omega} = \kappa (t_{\mathrm{c}} - t)^{-\xi}\text{, with } \xi > 0, \tag{S2}$$

where $\kappa = (\xi / \eta)^{\xi}$, $\xi = 1/(\alpha - 1)$, t is time, and t_{c} is the time of failure determined from the initial condition $\dot{\Omega}(t = t_0) = \dot{\Omega}_0$. Here, $\xi > 0$ (for $\alpha > 1$) ensures that $\dot{\Omega}$ exhibits a singular behavior at time $t = t_{\mathrm{c}}$.

An integration of equation (S2) leads to:

$$\Omega(t) = \begin{cases} A - \dfrac{\kappa}{m}(t_{\mathrm{c}} - t)^m, & m \neq 0 \\ A - \kappa \ln(t_{\mathrm{c}} - t), & m = 0 \end{cases}, \tag{S3}$$

where $m = 1 - \xi = 1 - 1/(\alpha - 1) < 1$ is a critical exponent and A can be determined from the initial condition of $\Omega(t = t_0) = \Omega_0$, so that:

$$\Omega(t) = \begin{cases} \Omega_0 + \dfrac{\kappa}{m}\left[(t_{\mathrm{c}} - t_0)^m - (t_{\mathrm{c}} - t)^m\right], & m \neq 0 \\ \Omega_0 - \kappa \ln\left(\dfrac{t_{\mathrm{c}} - t}{t_{\mathrm{c}} - t_0}\right), & m = 0 \end{cases}. \tag{S4}$$

Note that t_{c} is the time of failure determined from the other initial condition $\dot{\Omega}(t = t_0) = \dot{\Omega}_0$, giving $t_{\mathrm{c}} = t_0 + (\xi / \eta) \dot{\Omega}_0^{-1/\xi}$.

For $m \to 0$, consider the Taylor expansion $(t_{\mathrm{c}} - t_0)^m = e^{m \ln(t_{\mathrm{c}} - t_0)} = 1 + m \ln(t_{\mathrm{c}} - t_0) + O(m^2)$ and $(t_{\mathrm{c}} - t)^m = e^{m \ln(t_{\mathrm{c}} - t)} = 1 + m \ln(t_{\mathrm{c}} - t) + O(m^2)$, we have:

$$\Omega(t) = \Omega_0 + \frac{\kappa}{m}\left[e^{m \ln(t_{\mathrm{c}} - t_0)} - e^{m \ln(t_{\mathrm{c}} - t)}\right] = \Omega_0 - \kappa \ln\left(\frac{t_{\mathrm{c}} - t}{t_{\mathrm{c}} - t_0}\right) + O(m). \tag{S5}$$

Thus, as $m \to 0$, the solution of $\Omega(t)$ for $m \neq 0$ converges to that for $m = 0$.

Hence, by defining $B = -\kappa / m$, one can see that equation (1) in Methods gives the general solution of $\Omega(t)$ for $\alpha > 1$, $\xi > 0$, and thus $m < 1$ including $m = 0$, corresponding to an acceleration up to t_c.

Supplementary Note 2. Derivation of the log-periodic power law singularity (LPPLS) model

Let us define:

$$\tilde{\Omega}(t) = \frac{\Omega(t) - A}{B} = (t_c - t)^m , \tag{S6}$$

where $B \neq 0$ ensures the presence of a finite-time singularity. This power law relation obeys the symmetry of scale invariance, meaning that scaling $t_c - t$ by an arbitrary factor λ leads to a corresponding scaling of the observable by factor $\mu(\lambda)$, while the $t_c - t$ dependence remains unchanged. Mathematically, this is expressed as:

$$\mu\tilde{\Omega}(t) = \left[\lambda(t_c - t)\right]^m . \tag{S7}$$

In other words, if we replace $t_c - t$ with $\lambda(t_c - t)$ and replace $\tilde{\Omega}$ with $\mu\tilde{\Omega}$ in equation (S6), the equality still holds, reflecting the scale invariance.

From equations (S6) and (S7), we derive the following equation that determines $\mu(\lambda)$:

$$\lambda^m / \mu = 1. \tag{S8}$$

It is often the case that, rather than deriving $\mu(\lambda)$ from λ and m, first-principle considerations provide the values of λ and μ, from which one derives the value of the exponent as:

$$m = \frac{\ln \mu}{\ln \lambda} . \tag{S9}$$

In the presence of continuous scale invariance, the above derivation holds for arbitrary value of λ with $\mu(\lambda)$ adjusting so that there is a single value of the exponent m given by equation (S9). The general theoretical procedure is usually formulated with the Renormalization Group[5] by taking the limit $\lambda = 1 + \delta \to 1$ ($\delta \to 0$) and $\mu = 1 + \zeta \to 1$ ($\zeta \to 0$), so that $m = \delta / \zeta$ in the limit.

The continuous scale invariance discussed above can be partially broken into a discrete scale invariance[6]. This means that the power law relationship, equation (S7), holds under scaling $t_c - t$ by specific factors that are integer powers λ^n of a specific scaling ratio $\lambda > 1$, where n is an arbitrary integer indexing the hierarchy of scales[6]. There are several derivations of the

corresponding spectrum of power law exponents m_n. The simplest one is to start from the identity $1 = \exp(i2\pi n)$ and substitute it into the right-hand-side of equation (S8). Solving for m_n yields the series of complex critical exponents:

$$m_n = m + in\omega , \tag{S10}$$

where m is the real part given by equation (S9), n is an arbitrary integer, and $\omega = 2\pi / \ln \lambda$. The general form of $\tilde{\Omega}$ can be expressed as an infinite sum of power laws $(t_\mathrm{c} - t)^{m_n}$, similarly to a Fourier series — it is in fact a discrete Mellin transform generalization of discrete Fourier series[7]. The corresponding log-periodic power law is obtained by taking the real part of each power law $(t_\mathrm{c} - t)^{m_n}$, since observables are real. This gives:

$$\tilde{\Omega}_n(t) = \mathrm{Re}\left[(t_\mathrm{c} - t)^{m_n}\right] = (t_\mathrm{c} - t)^m \cos\left[n\omega \ln(t_\mathrm{c} - t)\right]. \tag{S11}$$

Thus, the most general solution of $\tilde{\Omega}$ is given by a superposition of all the components of this generalized Fourier series in logarithmic scale[8]:

$$\tilde{\Omega}(t) = (t_\mathrm{c} - t)^m \sum_{n=0}^{+\infty} a_n \cos\left[n\omega \ln(t_\mathrm{c} - t) - \phi_n\right], \tag{S12}$$

where a_n are the generalized Fourier coefficients and ϕ_n represent the phase shifts, with $a_0 = 1$ and $\phi_0 = 0$. The term for $n = 0$ defines a pure power law with fully continuous scale invariance governed by the real critical exponent m, while the terms for $n \geq 1$ introduce a partial breaking of continuous scale invariance into discrete scale invariance, resulting in a series of log-periodic oscillations decorating the pure power law[6]. It has been proven that the amplitudes of the coefficients a_n decay fast[7] with n, so that only the first correction term $n = 1$ is important in general (there are exceptions in which higher-order terms need to be considered but we do not consider this situation here). Keeping the terms $n = 0$ and $n = 1$ in equation (S12) recovers the LPPLS formula, i.e., equation (2) in the Methods.

The local maxima of the log-periodic term $C\cos\left[\omega \ln(t_\mathrm{c} - t) - \phi\right]$ in the LPPLS formula occur at times converging to t_c according to a geometric time series $\{t_1, t_2, \ldots, t_k, \ldots\}$ with $t_\mathrm{c} - t_k = \rho\lambda^{-k}$, where k is an integer and $\rho = \exp(\phi / \omega)$ is a time constant determined from the initial conditions. The geometric time series corresponds to the times for which the argument of the cosine function is an integer multiple of 2π.

Supplementary Note 3. Global dataset of geohazard events and data acquisition approach

In this study, we have compiled a large global dataset of 109 historical geohazard events of landslides, rockbursts, glacier breakoffs, and volcanic eruptions across seven continents over the past century (see Supplementary Tables 1-4). These data were retrieved through two major ways: (1) exported directly from the monitoring system and obtained from either published dataset/database or from the authors (indicated as "Original" in Supplementary Tables 1-4), and (2) digitized from figures in published literature using digitization software (indicated as "Digitized" in Supplementary Tables 1-4).

Note that the data of 10 cases (Agoyama, Arvigo, Galterengraben, Grabengufer, Hogarth, Kagemori, La Saxe, Nevis Bluff, Vajont, and Weissmiess) were extracted from the published dataset (https://mediatum.ub.tum.de/1688868)[9], where the data of 4 cases are "Original" and the data of other 6 cases are "Digitized". Most volcano data are downloaded from the WOVOdat platform (https://www.wovodat.org), which is a publicly accessible database of volcanic unrest[10]. For most of digitized data in our dataset, we employ the software PlotDigitizer Pro (https://plotdigitizer.com) to retrieve the data from the published literature. The references for all the data are indicated in Supplementary Tables 1-4.

Supplementary Note 4. Calibration of the LPPLS model

For the time series of N measurements of the observable quantity $\mathbf{\Omega} = \{\Omega_1, \Omega_2, ..., \Omega_N\}$ recorded at time $\mathbf{t} = \{t_1, t_2, ..., t_N\} \in [\tau, T]$ (τ and T respectively denote the start and end of the time window over which the fitting is performed), the LPPLS model is calibrated based on the following scheme[11].

The LPPLS parameter set $\mathbf{\theta}_{\mathrm{LPPLS}} = \{A, B, C, t_\mathrm{c}, m, \omega, \phi\}$ has seven parameters with the former three being linear and the latter four being nonlinear. By introducing $C_1 = C\cos\phi$ and $C_2 = C\sin\phi$, we can rewrite the original LPPLS formula, i.e., equation (2) in Methods, as:

$$\Omega(t) = A + B(t_\mathrm{c} - t)^m + C_1(t_\mathrm{c} - t)^m \cos\left[\omega \ln(t_\mathrm{c} - t)\right] + C_2(t_\mathrm{c} - t)^m \sin\left[\omega \ln(t_\mathrm{c} - t)\right], \quad \text{(S13)}$$

where the new parameter set $\mathbf{\theta}_{\mathrm{LPPLS}} = \{A, B, C_1, C_2, t_\mathrm{c}, m, \omega\}$ still has seven parameters but now with four linear and only three nonlinear parameters. To estimate all these parameters, we define the cost function as the sum of squared errors:

$$F(\mathbf{\theta}_{\mathrm{LPPLS}}; \mathbf{\Omega}, \mathbf{t}) = \sum_{i=1}^{N} \varepsilon_i^2 , \quad \text{(S14)}$$

with each residual calculated as:

$$\varepsilon_i = \Omega_i - A - B(t_c - t_i)^m - C_1(t_c - t_i)^m \cos\left[\omega \ln(t_c - t_i)\right] - C_2(t_c - t_i)^m \sin\left[\omega \ln(t_c - t_i)\right]. \quad \text{(S15)}$$

The ordinary least squares method amounts to minimizing the cost function to obtain the estimates for the model parameters:

$$\hat{\boldsymbol{\theta}}_{\text{LPPLS}} = \arg\min_{\boldsymbol{\theta}_{\text{LPPLS}}} F(\boldsymbol{\theta}_{\text{LPPLS}}; \boldsymbol{\Omega}, \mathbf{t}) . \quad \text{(S16)}$$

This is not a trivial task due to the strong nonlinearity of the cost function and the presence of multiple local minima.

To solve this minimization problem, we enslave the four linear parameters $\{A, B, C_1, C_2\}$ to the three nonlinear ones $\{t_c, m, \omega\}$ so as to reduce the minimization problem to:

$$\{\hat{t}_c, \hat{m}, \hat{\omega}\} = \arg\min_{t_c, m, \omega} F_1(t_c, m, \omega) , \quad \text{(S17)}$$

with the profiled cost function defined as:

$$F_1(t_c, m, \omega) = \min_{A,B,C_1,C_2} F(A, B, C_1, C_2, t_c, m, \omega) = F(t_c, m, \omega, \hat{A}, \hat{B}, \hat{C}_1, \hat{C}_2) , \quad \text{(S18)}$$

and the estimates for the linear parameters $\{A, B, C_1, C_2\}$ obtained by solving the optimization problem for fixed values of the nonlinear parameters $\{t_c, m, \omega\}$:

$$\{\hat{A}, \hat{B}, \hat{C}_1, \hat{C}_2\} = \arg\min_{A,B,C_1,C_2} F(A, B, C_1, C_2, t_c, m, \omega) , \quad \text{(S19)}$$

which has a unique solution analytically solved from the following system of linear equations:

$$\begin{bmatrix} N & \sum f_i & \sum g_i & \sum h_i \\ \sum f_i & \sum f_i^2 & \sum f_i g_i & \sum f_i h_i \\ \sum g_i & \sum f_i g_i & \sum g_i^2 & \sum g_i h_i \\ \sum h_i & \sum f_i h_i & \sum g_i h_i & \sum h_i^2 \end{bmatrix} \begin{bmatrix} \hat{A} \\ \hat{B} \\ \hat{C}_1 \\ \hat{C}_2 \end{bmatrix} = \begin{bmatrix} \sum \Omega_i \\ \sum \Omega_i f_i \\ \sum \Omega_i g_i \\ \sum \Omega_i h_i \end{bmatrix} , \quad \text{(S20)}$$

where $f_i = (t_c - t_i)^m$, $g_i = (t_c - t_i)^m \cos\left[\omega \ln(t_c - t_i)\right]$, and $h_i = (t_c - t_i)^m \sin\left[\omega \ln(t_c - t_i)\right]$.

The optimization problem, equation (S17), can be further reformulated as:

$$\hat{t}_c = \arg\min_{t_c} F_2(t_c) , \quad \text{(S21)}$$

with the cost function given by:

$$F_2(t_c) = \min_{m,\omega} F_1(t_c, m, \omega) = F_1(t_c, \hat{m}, \hat{\omega}) , \quad \text{(S22)}$$

and the estimates for parameters $\{m, \omega\}$ obtained by solving the optimization problem:

$$\{\hat{m}, \hat{\omega}\} = \arg\min_{m,\omega} F_1(t_c, m, \omega)\,. \tag{S23}$$

Here, a constraint of $4.94 \le \omega \le 15$ is imposed with the lower bound defined to prevent chaotic scenarios[12] and the upper bound defined to avoid spurious oscillations[11], so that the scaling ratio $\lambda = \exp(2\pi / \omega)$ is at the order[6] of 2, as suggested by general theoretical arguments[13].

Implementing the LPPLS calibration algorithm involves the following steps. First, a fixed grid $t_{c,j}$ (with $j = 1, 2, ..., n_{tc}$ and n_{tc} being the number of grid points) is defined to scan t_c over a prescribed range $[T, T + \delta(T - \tau)]$ (with τ and T being respectively the start and end of the time window in which the calibration is performed), where δ (set to 0.1 in our code) controls the horizon of extrapolation beyond the fitting window during the initial fixed-grid search. Second, for each grid point $t_{c,j}$, we solve the optimization problem of equation (S23) to estimate parameters $\{m_j, \omega_j\}$ based on the Nelder-Mead simplex search method, with the enslaved linear parameters $\{A_j, B_j, C_{1,j}, C_{2,j}\}$ determined from equations (S19) and (S20) via a subroutine embedded within the optimization procedure. Then, the optimal $t_{c,j*}$ (with $j*$ indicating the index of the optimal $t_{c,j}$ within the fixed grid) is determined by equation (S21), by comparing the costs given by equation (S22) obtained at all the grid points. Lastly, the optimal nonlinear parameter set $\{t_{c,j*}, m_{j*}, \omega_{j*}\}$ is used as the initial guess for the final optimization using the Nelder-Mead simplex search method, where t_c is searched freely within the interval of $[t_{c,j*}, t_{c,j*+1})$ with the right bound being infinity if $j* = n_{tc}$, while the enslaved linear parameters $\{A, B, C_1, C_2\}$ are calculated analytically by equation (S20) via a subroutine embedded within the optimization procedure.

Supplementary Note 5. Calibration of the PLS model

For the time series of N measurements of the observable quantity $\mathbf{\Omega} = \{\Omega_1, \Omega_2, ..., \Omega_N\}$ recorded at time $\mathbf{t} = \{t_1, t_2, ..., t_N\} \in [\tau, T]$ (τ and T respectively denote the start and end of the time window over which the fitting is performed), the PLS model is calibrated based on a scheme similar to the one for the LPPLS model.

The PLS model, i.e., equation (1) in Methods, has a parameter set $\boldsymbol{\theta}_{PLS} = \{A, B, t_c, m\}$ including four parameters with the first two being linear and the last two being nonlinear. To estimate all these parameters, we define the cost function as the sum of squared errors:

$$F(\boldsymbol{\theta}_{\mathrm{PLS}};\boldsymbol{\Omega},\mathbf{t})=\sum_{i=1}^{N}\varepsilon_i^2\,, \tag{S24}$$

with each residual calculated as:

$$\varepsilon_i=\Omega_i-A-B(t_{\mathrm{c}}-t_i)^m\,. \tag{S25}$$

The ordinary least squares method amounts to minimizing the cost function to obtain the estimates for the model parameters:

$$\hat{\boldsymbol{\theta}}_{\mathrm{PLS}}=\arg\min_{\boldsymbol{\theta}_{\mathrm{PLS}}}F(\boldsymbol{\theta}_{\mathrm{PLS}})\,. \tag{S26}$$

To do so, we enslave the two linear parameters $\{A,B\}$ to the two nonlinear ones $\{t_{\mathrm{c}},m\}$ to obtain the nonlinear optimization problem:

$$\{\hat{t}_{\mathrm{c}},\hat{m}\}=\arg\min_{t_{\mathrm{c}},m}F_1(t_{\mathrm{c}},m)\,, \tag{S27}$$

with the cost function defined as:

$$F_1(t_{\mathrm{c}},m)=\min_{A,B}F(t_{\mathrm{c}},m,A,B)=F(t_{\mathrm{c}},m,\hat{A},\hat{B})\,. \tag{S28}$$

The estimates for parameters $\{A,B\}$ are obtained by solving the optimization problem:

$$\{\hat{A},\hat{B}\}=\arg\min_{A,B}F(t_{\mathrm{c}},m,A,B)\,, \tag{S29}$$

which has a unique solution analytically solved from the following system of linear equations:

$$\begin{bmatrix} N & \sum f_i \\ \sum f_i & \sum f_i^2 \end{bmatrix}\begin{bmatrix}\hat{A}\\ \hat{B}\end{bmatrix}=\begin{bmatrix}\sum\Omega_i\\ \sum\Omega_i f_i\end{bmatrix}, \tag{S30}$$

where $f_i=(t_{\mathrm{c}}-t_i)^m$. The optimization problem, equation (S27), can be further reformulated as:

$$\hat{t}_{\mathrm{c}}=\arg\min_{t_{\mathrm{c}}}F_2(t_{\mathrm{c}})\,, \tag{S31}$$

with the cost function given by:

$$F_2(t_{\mathrm{c}})=\min_{m}F_1(t_{\mathrm{c}},m)=F_1(t_{\mathrm{c}},\hat{m})\,, \tag{S32}$$

and the estimate for parameter *m* is obtained by solving the optimization problem:

$$\hat{m}=\arg\min_{m}F_1(t_{\mathrm{c}},m)\,. \tag{S33}$$

Implementing the PLS calibration algorithm involves the following steps. First, a fixed grid $t_{\mathrm{c},j}$ (with $j=1,2,...,n_{\mathrm{tc}}$ and n_{tc} being the number of grid points) is defined to scan t_{c} over a prescribed range $[T,T+\delta(T-\tau)]$ (with τ and T being respectively the start and end of the time

window in which the calibration is performed), where δ (set to 0.1 in our code) controls the degree of extrapolation beyond the fitting window during the initial fixed-grid search. Second, for each grid point $t_{c,j}$, we solve the optimization problem of equation (S33) to estimate parameters m_j based on the Nelder-Mead simplex search method, with the enslaved linear parameters $\{A_j, B_j\}$ determined from equations (S29) and (S30) via a subroutine embedded within the optimization procedure. Then, the optimal t_{c,j^*} (with j^* indicating the index of the optimal $t_{c,j}$ within the fixed grid) is determined by equation (S31), by comparing the costs given by equation (S32) obtained at all the grid points. Lastly, the optimal nonlinear parameter set $\{t_{c,j^*}, m_{j^*}\}$ is used as the initial guess for the final optimization using the Nelder-Mead simplex search method, where t_c is searched freely within the interval of $[t_{c,j^*}, t_{c,j^*+1})$ with the right bound being infinity if $j^* = n_{tc}$, while the enslaved linear parameters $\{A, B\}$ are calculated analytically by equation (S30) via a subroutine embedded within the optimization procedure.

Supplementary Note 6. Lagrange regularization approach

For a fixed end time T, the optimal start time τ of the time window for calibrating the LPPLS or PLS model can be endogenously detected using the Lagrange regularization approach[14] with the following cost function to minimize:

$$\tilde{F}'(\tau) = \tilde{F}(\tau) - \chi N(\tau), \tag{S34}$$

where χ is the Lagrange parameter, N is the number of observations within the time window $[\tau, T]$, and $\tilde{F}(\tau)$ is the normalized sum of squared residuals given by:

$$\tilde{F}(\tau) = \frac{F}{N(\tau) - r}, \tag{S35}$$

where F is the sum of squared errors given by equation (S14) for the LPPLS fitting or by equation (S24) for the PLS fitting and r is the number of degrees of freedom of the model (i.e., 7 and 4 for the LPPLS and PLS models, respectively). The Lagrange parameter χ can be heuristically approximated by the slope in the linear regression model of $\tilde{F}(\tau)$ with respect to τ.

When comparing the LPPLS and PLS models, we estimate τ based on the LPPLS model to determine the time window for comparison. Alternatively, we can estimate τ based on the PLS model or as the maximum of the τ values from the LPPLS and PLS models, which do not affect our conclusions about the model's superiority.

Supplementary Note 7. Log-likelihood function

In the context of ordinary least squares, if the model is well-specified (i.e., it represents the true generative process of the data, with the noise being independent and identically distributed), then the error term ε obeys a zero-mean Gaussian distribution:

$$f(\varepsilon;\sigma^2) = \frac{1}{\sqrt{2\pi\sigma^2}} \exp\left(-\frac{\varepsilon^2}{2\sigma^2}\right), \tag{S36}$$

where σ^2 is the variance. We construct the likelihood function as:

$$L(\boldsymbol{\theta},\sigma^2;\boldsymbol{\Omega},\mathbf{t}) = \prod_{i=1}^{N}\left[\frac{1}{\sqrt{2\pi\sigma^2}}\exp\left(-\frac{\varepsilon_i^2}{2\sigma^2}\right)\right] = (2\pi\sigma^2)^{-N/2}\exp\left[-\frac{F(\boldsymbol{\theta};\boldsymbol{\Omega},\mathbf{t})}{2\sigma^2}\right]. \tag{S37}$$

The corresponding log-likelihood function is:

$$\ln L(\boldsymbol{\theta},\sigma^2;\boldsymbol{\Omega},\mathbf{t}) = -\frac{N}{2}\ln(2\pi\sigma^2) - \frac{F(\boldsymbol{\theta};\boldsymbol{\Omega},\mathbf{t})}{2\sigma^2}, \tag{S38}$$

with the maximum likelihood estimate for σ^2 given as:

$$\hat{\sigma}^2 = \frac{1}{N}F(\hat{\boldsymbol{\theta}};\boldsymbol{\Omega},\mathbf{t}), \tag{S39}$$

so that we obtain the log-likelihood function as:

$$\ln L(\hat{\boldsymbol{\theta}};\boldsymbol{\Omega},\mathbf{t}) = -\frac{N}{2}\left[\ln F(\hat{\boldsymbol{\theta}};\boldsymbol{\Omega},\mathbf{t}) + \ln\left(\frac{2\pi}{N}\right) + 1\right]. \tag{S40}$$

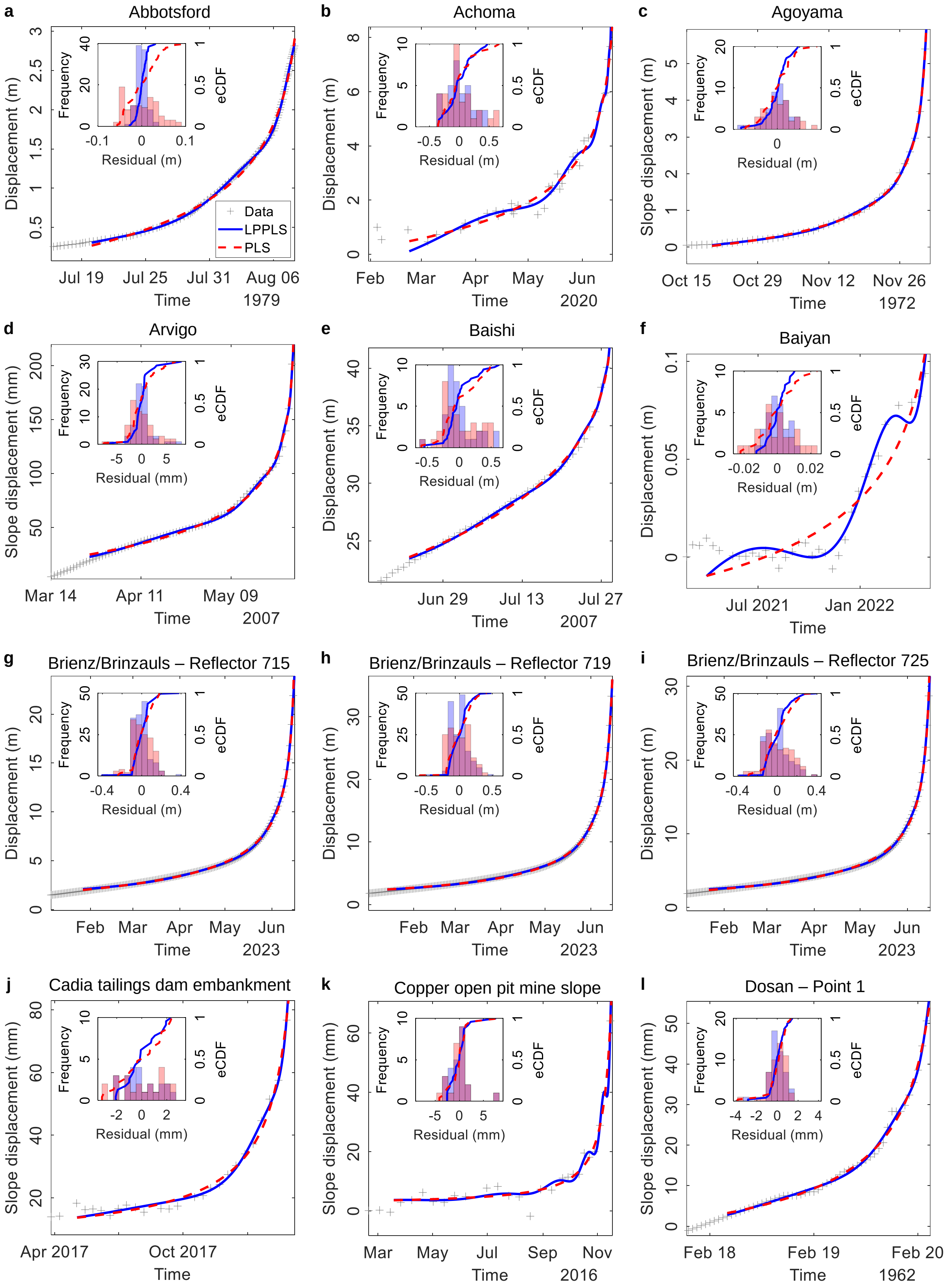

Supplementary Fig. 1. Comparison of the LPPLS and PLS models in fitting landslide data.

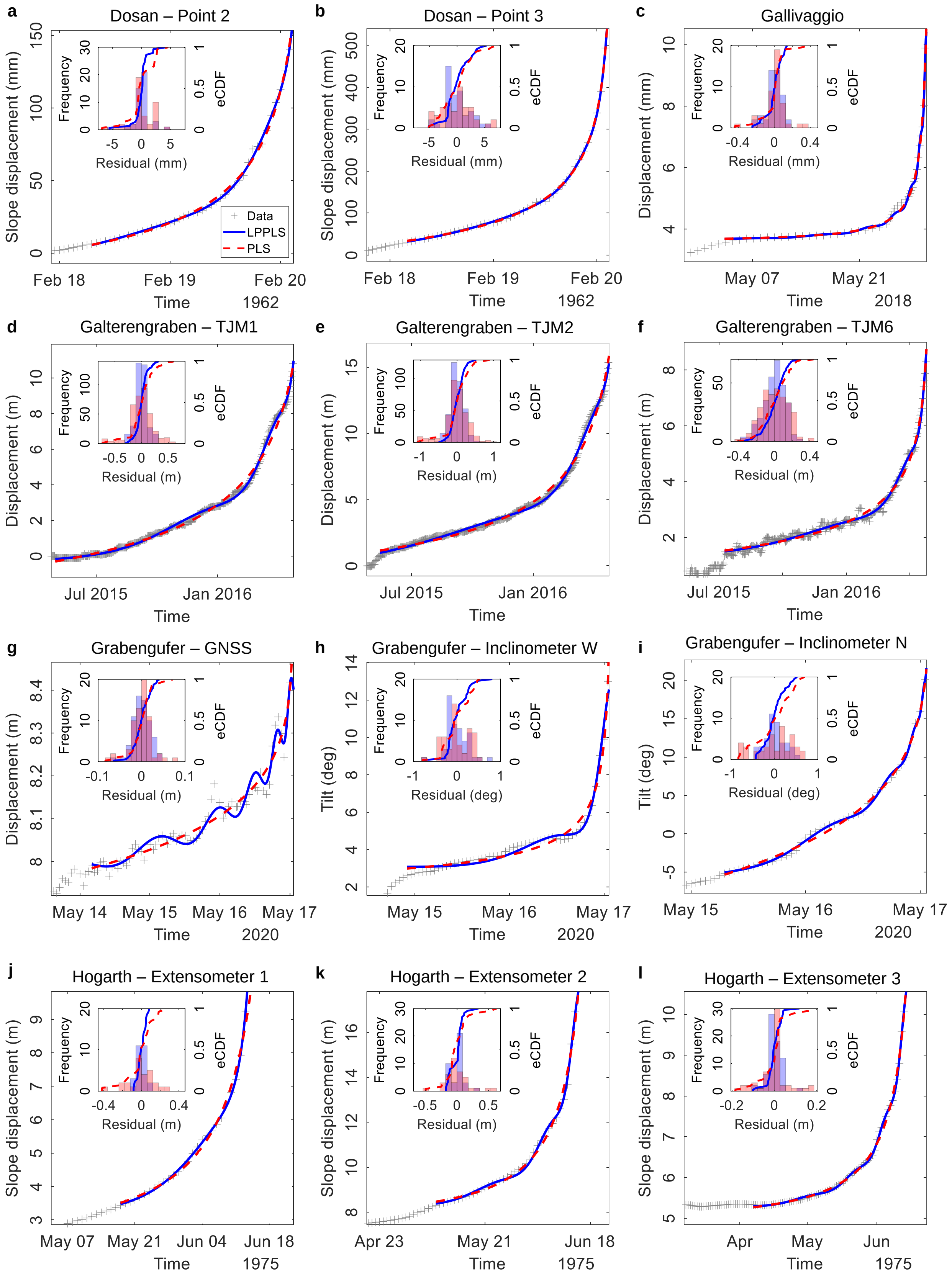

Supplementary Fig. 2. Comparison of the LPPLS and PLS models in fitting landslide data.

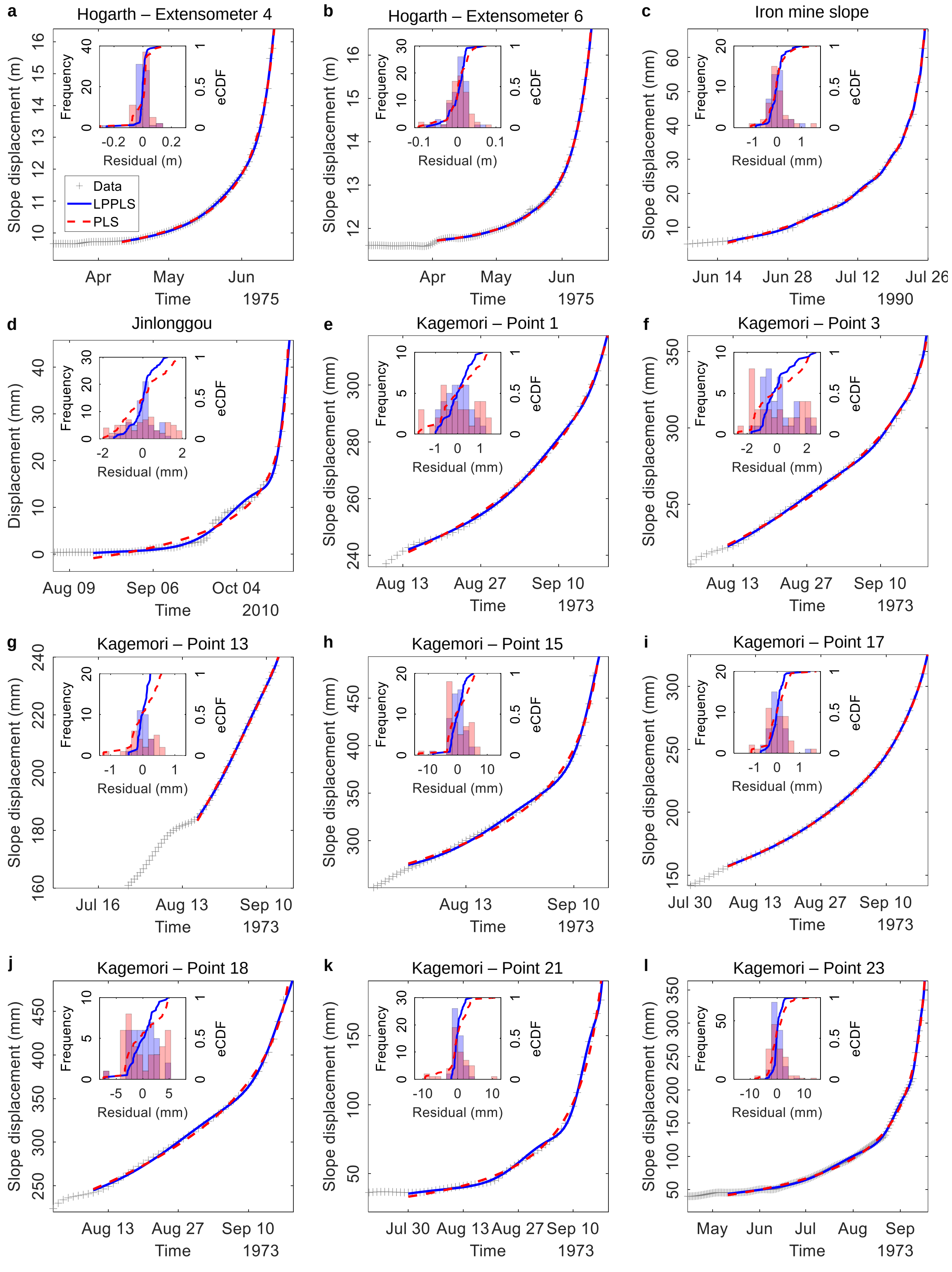

Supplementary Fig. 3. Comparison of the LPPLS and PLS models in fitting landslide data.

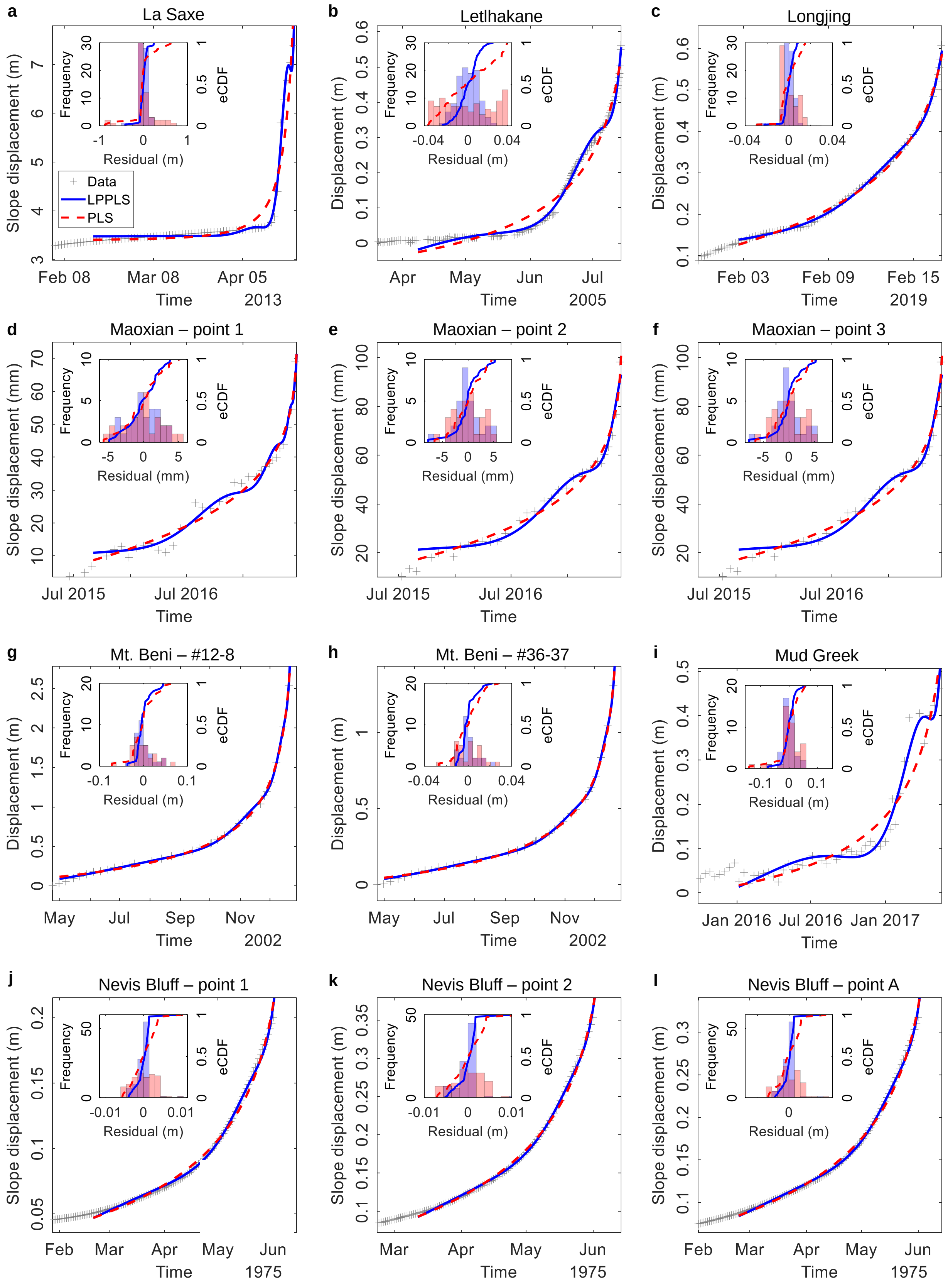

Supplementary Fig. 4. Comparison of the LPPLS and PLS models in fitting landslide data.

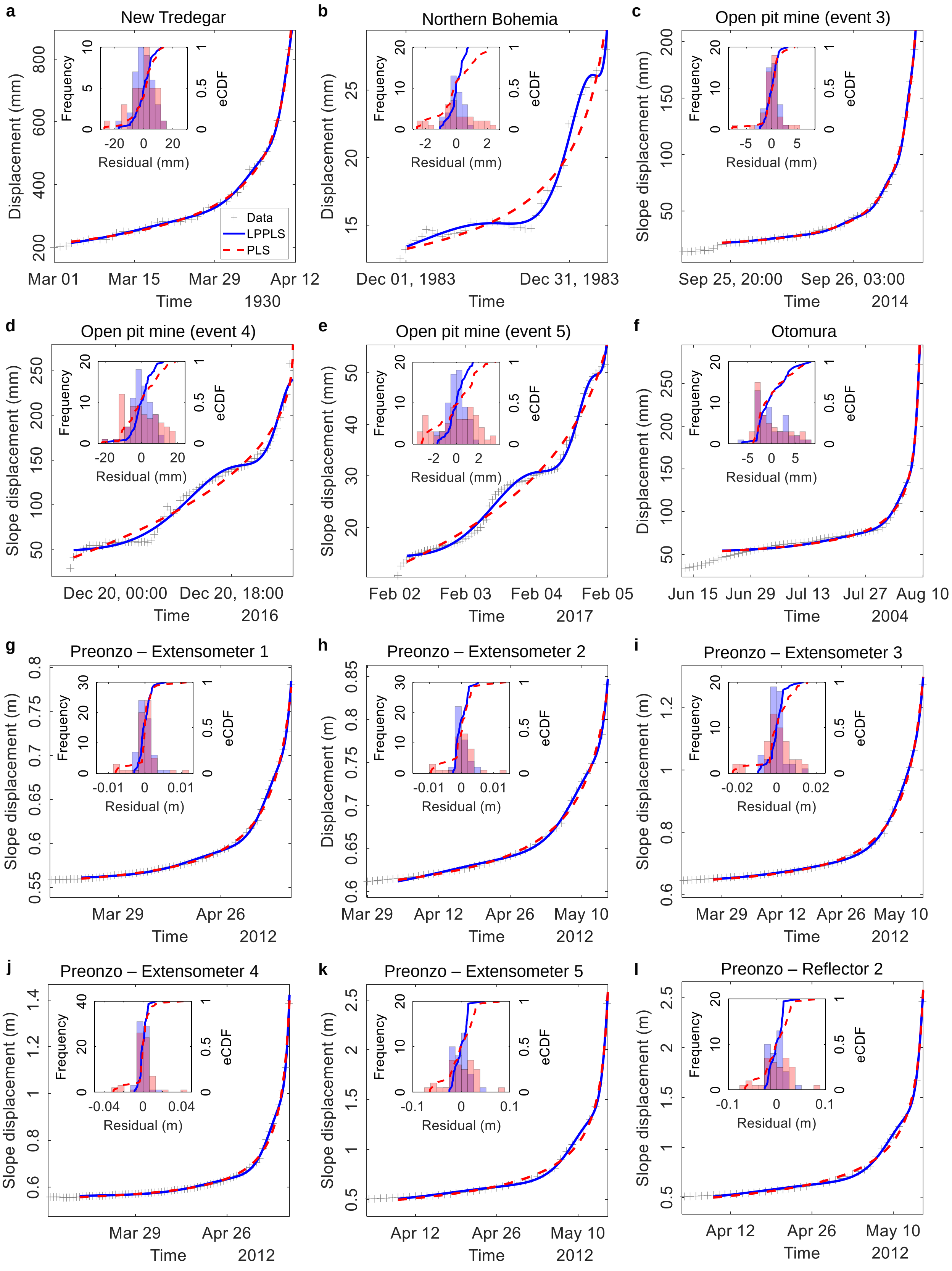

Supplementary Fig. 5. Comparison of the LPPLS and PLS models in fitting landslide data.

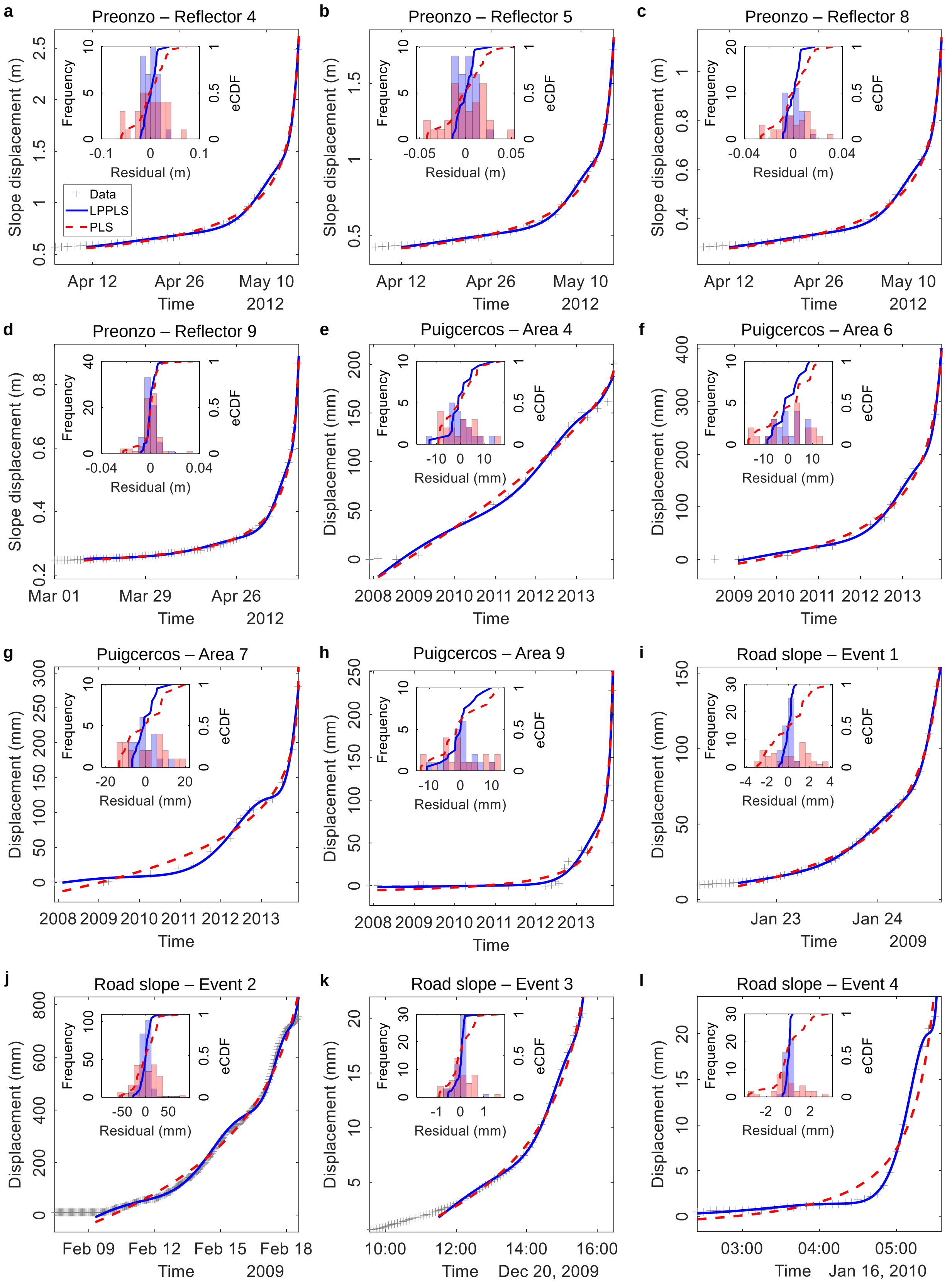

Supplementary Fig. 6. Comparison of the LPPLS and PLS models in fitting landslide data.

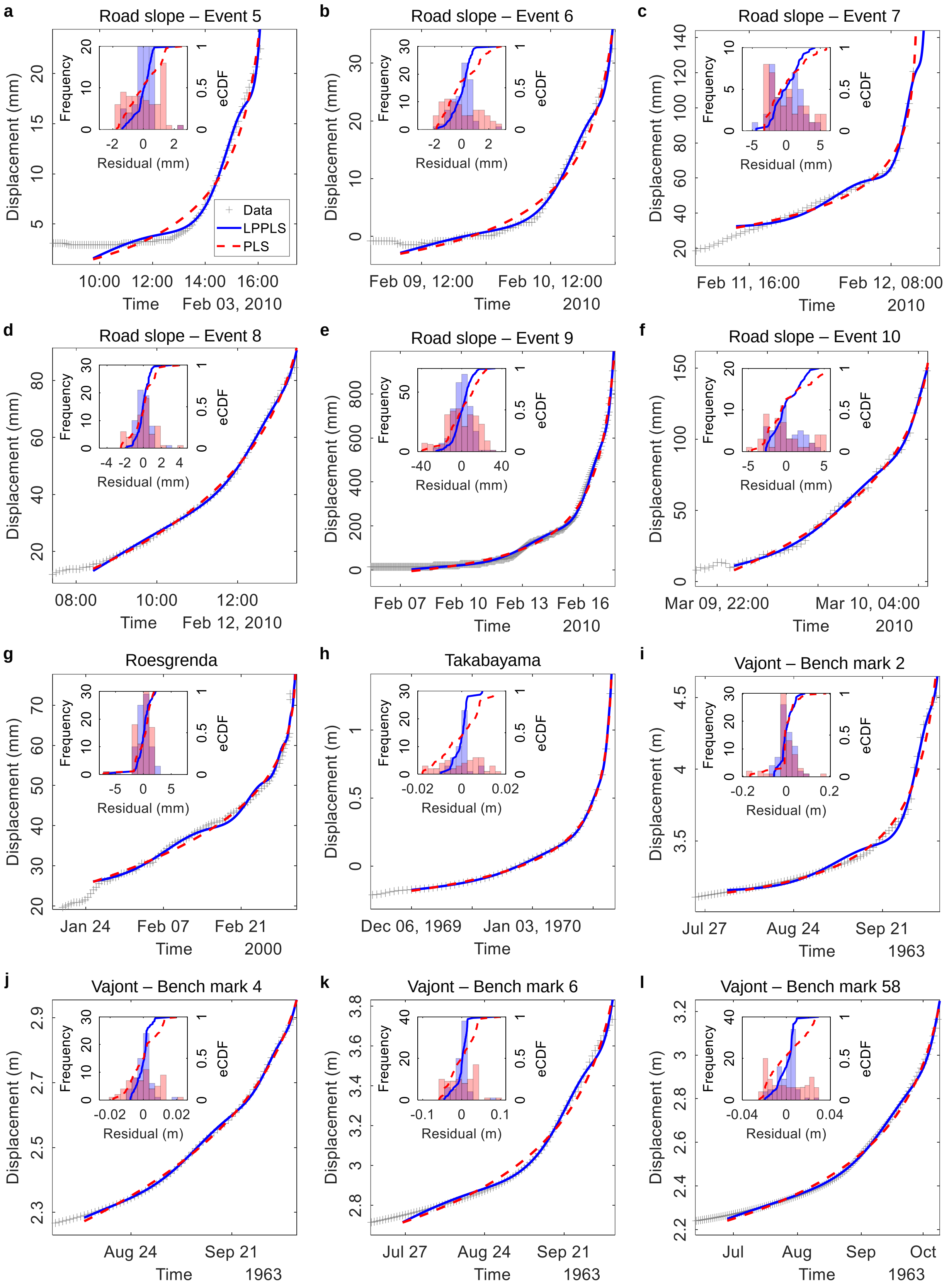

Supplementary Fig. 7. Comparison of the LPPLS and PLS models in fitting landslide data.

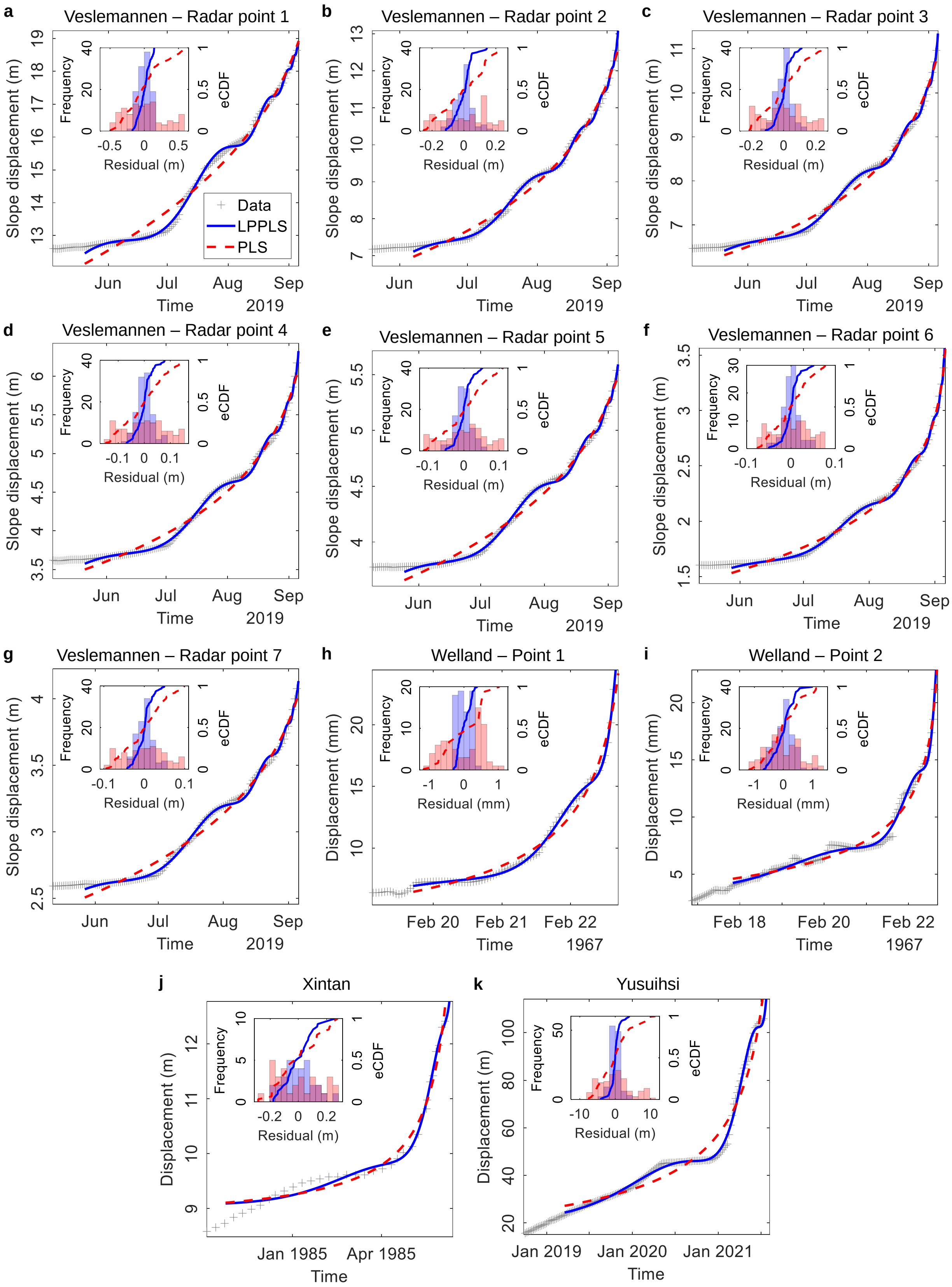

Supplementary Fig. 8. Comparison of the LPPLS and PLS models in fitting landslide data.

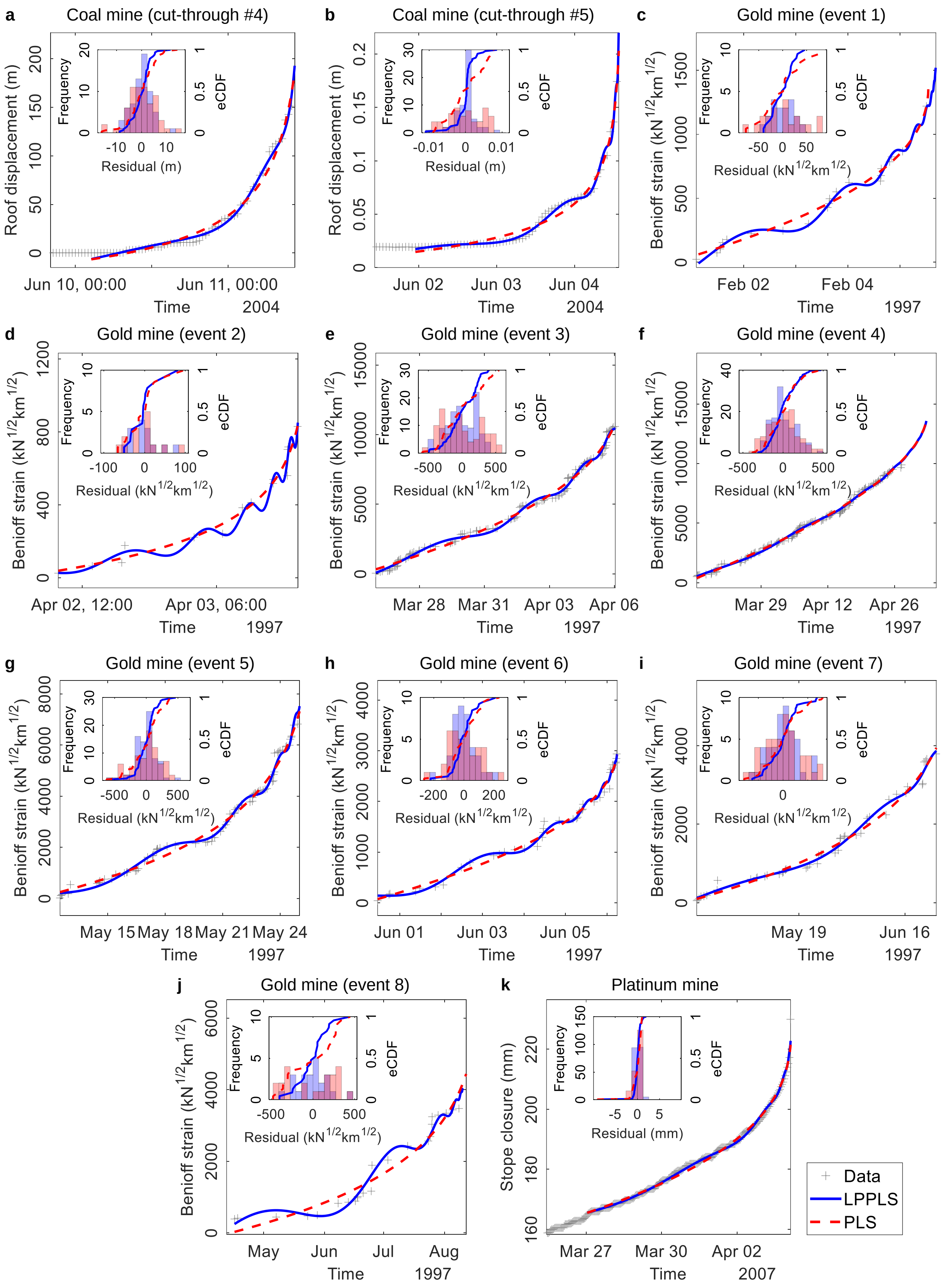

Supplementary Fig. 9. Comparison of the LPPLS and PLS models in fitting rockburst data.

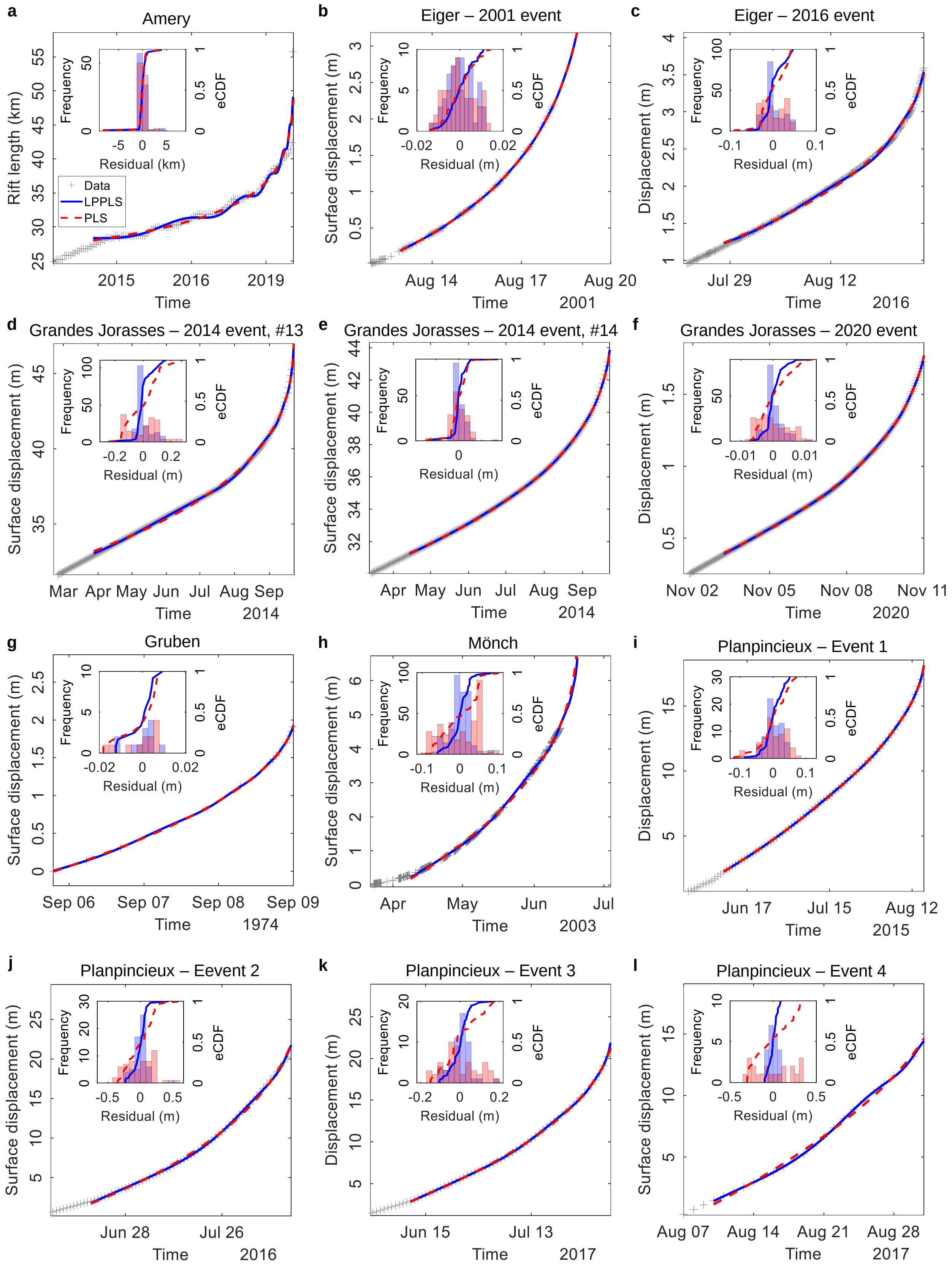

Supplementary Fig. S10. Comparison of the LPPLS and PLS models in fitting glacier data.

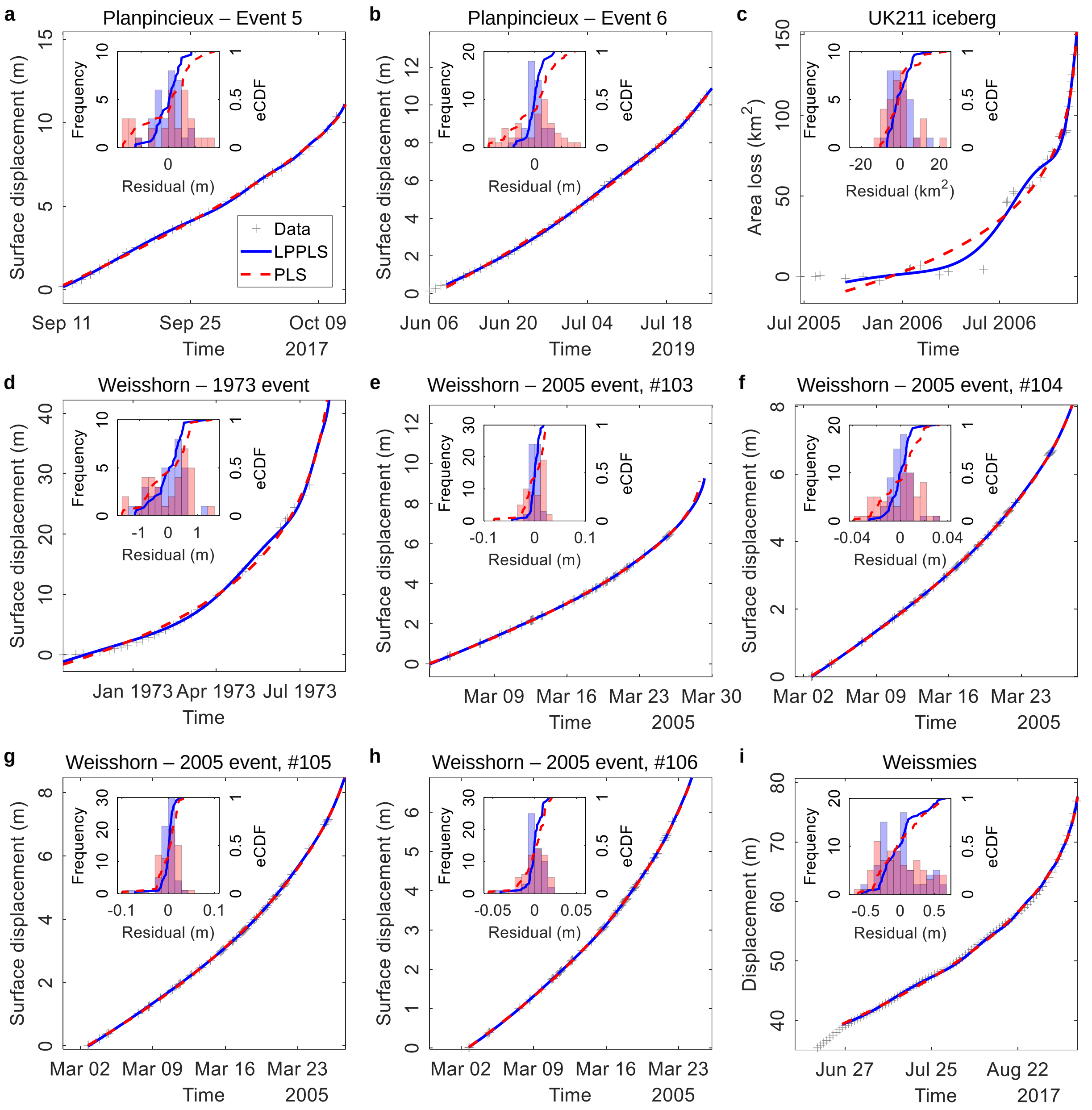

Supplementary Fig. 11. Comparison of the LPPLS and PLS models in fitting glacier data.

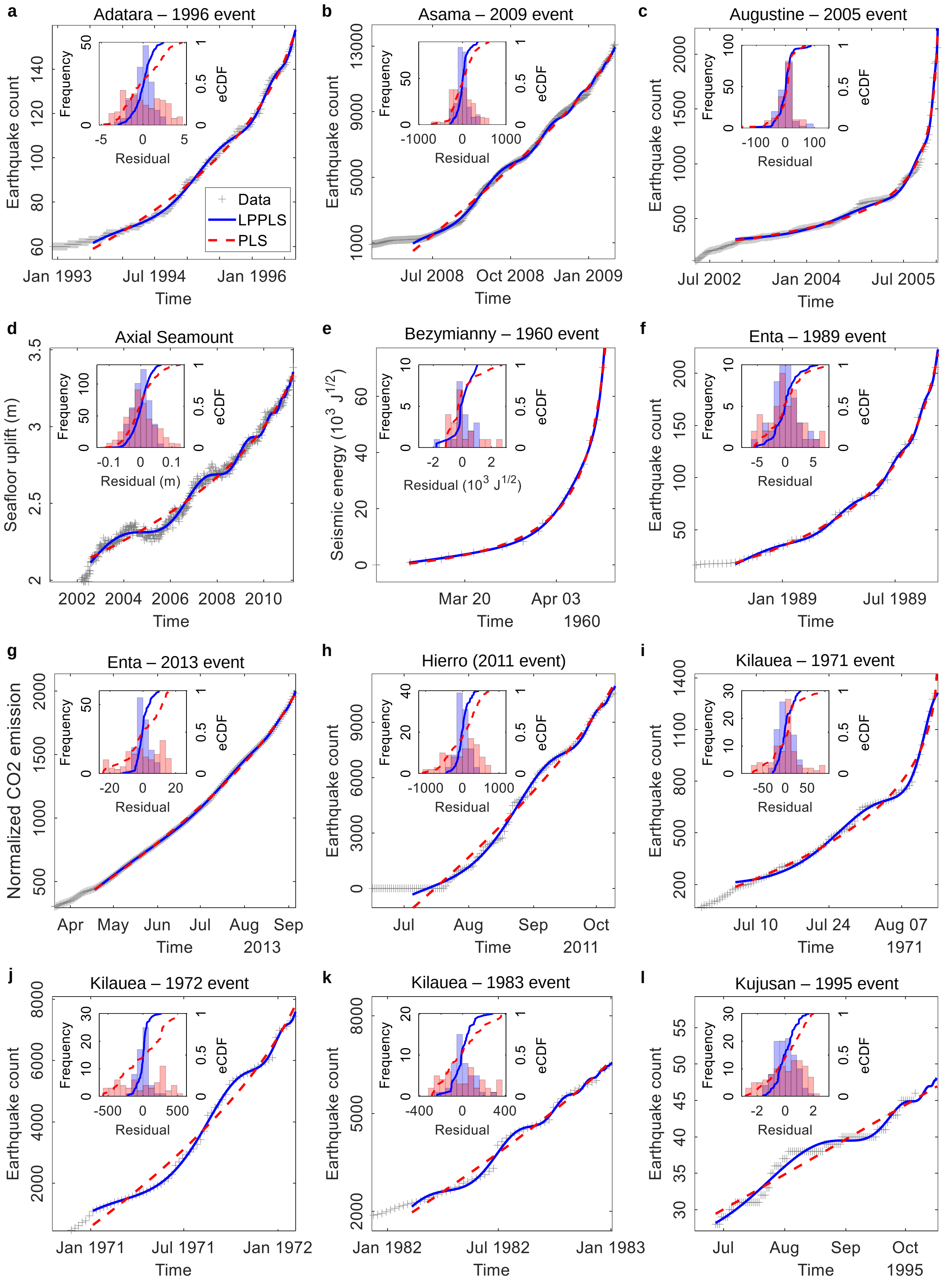

Supplementary Fig. 12. Comparison of the LPPLS and PLS models in fitting volcano data.

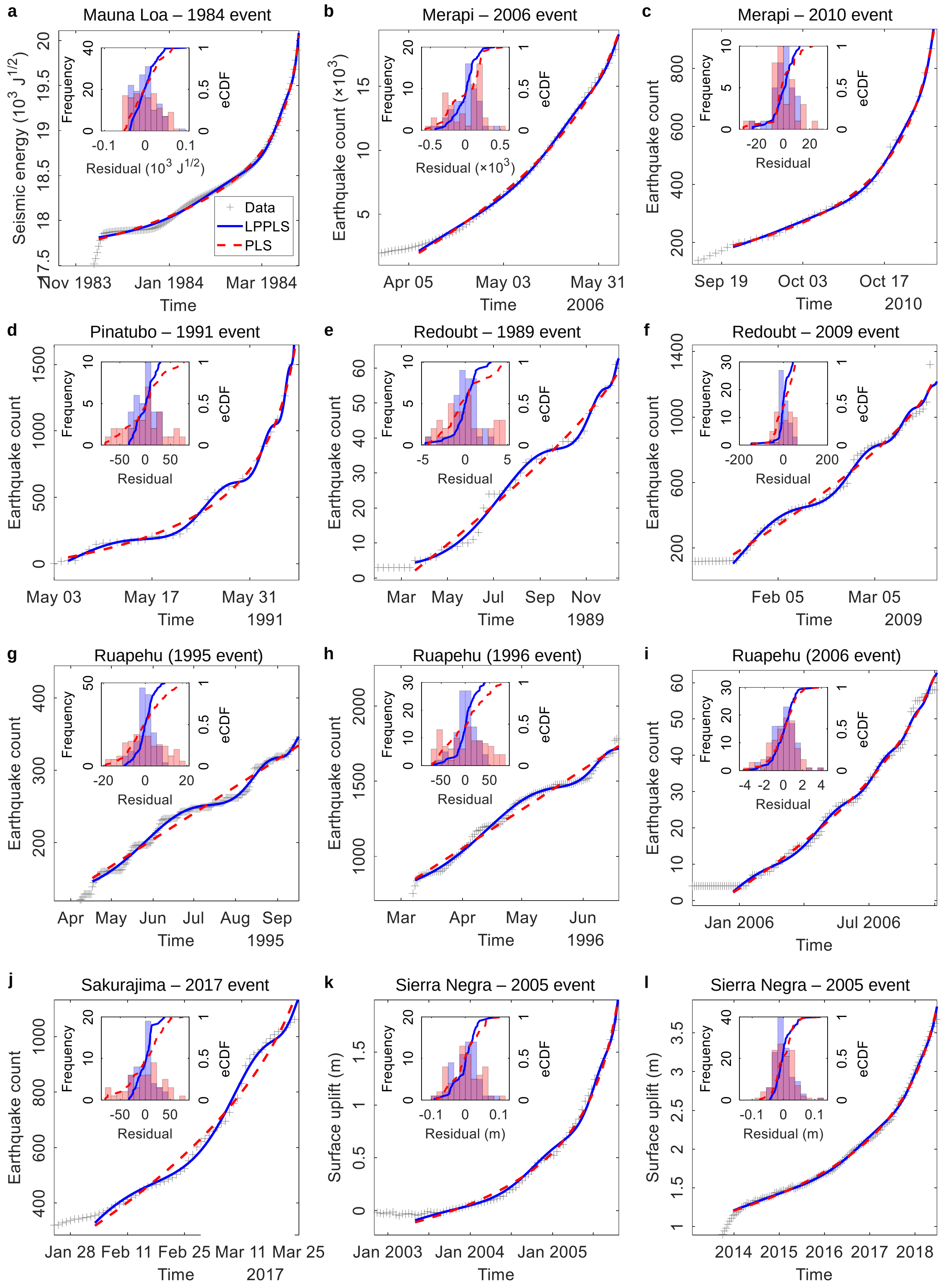

Supplementary Fig. 13. Comparison of the LPPLS and PLS models in fitting volcano data.

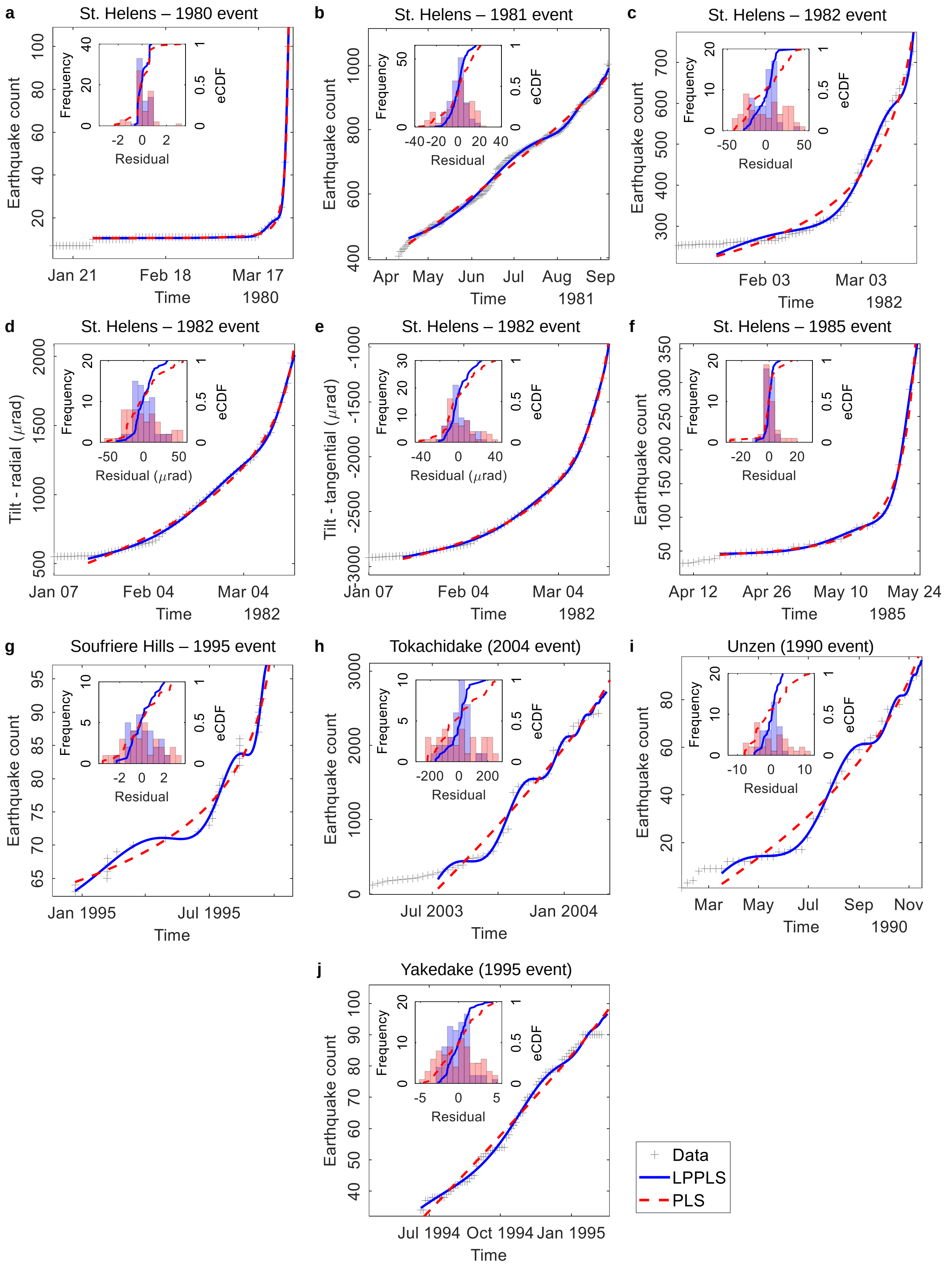

Supplementary Fig. 14. Comparison of the LPPLS and PLS models in fitting volcano data.

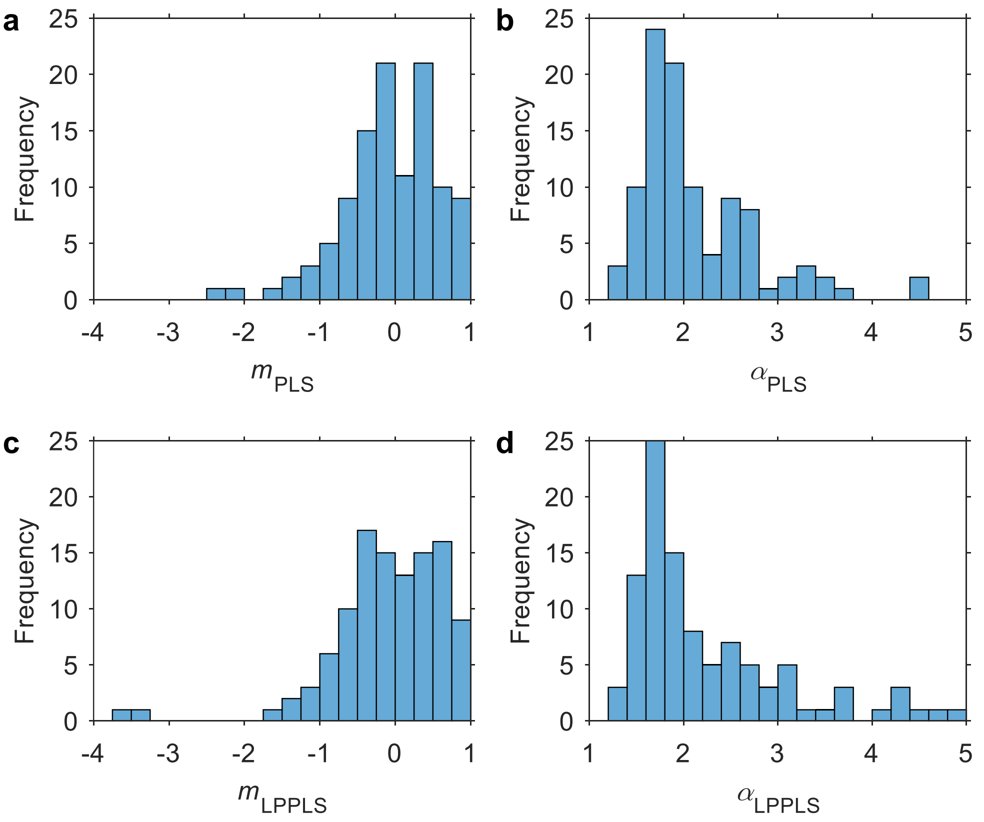

Supplementary Fig. 15. Histograms of the critical exponent *m* and nonlinearity exponent *α* of the PLS and LPPLS models for 109 geohazard events.

Supplementary Table 1. Landslide cases (49 events in total).

Site	Location	Type	Material	Failure time	Volume (m^3)	Monitoring method	Data source	Reference
Abbotsford	New Zealand	Soilslide	Clay	1979-08-08	5×10^6	Survey lines	Digitized	15
Achoma	Peru	Rock-soilslide	Lacustrine sediments	2020-06-16	5.4×10^6	Optical satellites	Digitized	16
Agoyama	Japan	Rockslide	Tuffaceous sandstone	1972-12-02	$\sim10^5$	Geodetic bench marks	Digitized	17
Arvigo	Switzerland	Topple / rockslide	Gneiss	2007-05-28	2×10^5	Telejointmeter	Original	9
Baishi	China	Rockslide	Phyllite	2007-07-28	2×10^6	Total station with reflectors	Digitized	18
Baiyan	China	Rockslide	Limestone	2022-05-08	2.5×10^4	Satellite-based InSAR	Digitized	19
Brienz / Brinzauls	Switzerland	Rockslide	Flysch, schists, dolomite	2023-06-15	1.2×10^6	Total station with reflectors	Original	20
Cadia	Australia	Soilslide	Earthfill materials	2018-03-09	7.2×10^4	Satellite-based InSAR	Digitized	21
Copper open pit	Undisclosed	Rockslide	Limestone, spilite	2016-11-17	6.4×10^5	Satellite-based InSAR	Digitized	21
Dosan	Japan	Rockslide	Schist	1962-02-20	6×10^4	Crack meter	Digitized	22
Gallivaggio	Italy	Rockfall	Granite	2018-05-29	5×10^3	Ground-based InSAR	Digitized	23
Galterengraben	Switzerland	Rockfall	Sandstone	2016-04-24	2.5×10^3	Telejointmeter	Original	9
Grabengufer	Switzerland	Rockfall	Rock & ice	2020-05-17	5×10^2	GNSS & inclinometer	Original	24
Hogarth	Canada	Topple	Diorite	1975-06-23	2×10^5	Extensometers	Digitized	25
Iron mine	Mexico	Rockslide	Rock	1990-07-26	$\sim5\times10^4$	Total station with reflectors	Digitized	26
Jinlonggou	China	Rockslide	Syenite & basalt	2010-10-23	2×10^5	Extensometer	Digitized	27
Kagemori	Japan	Rockslide	Limestone	1973-09-20	3×10^5-4×10^5	Measuring tapes	Digitized	28
La Saxe	Italy	Rockslide	Meta-sedimentary sequences	2013-04-21	5×10^2-1×10^3	Total station with reflectors	Original	29
Letlhakane diamond mine	Botswana	Rockslide	Sandstone	2005-07-14	2.3×10^5	Total station with reflectors	Digitized	30
Longjing	China	Rockslide	Dolomite & limestone	2019-02-17	1.4×10^6	Extensometers	Digitized	31
Maoxian	China	Soilslide	Soil	2017-06-24	1.5×10^7	Satellite-based InSAR	Digitized	32
Mt. Beni	Italy	Rockslide	Basalt & limestone	2002-12-28	5.0×10^5	Distometric benchmarks	Digitized	33

Supplementary Table 1 (continued). Landslide cases (49 events in total).

Site	Location	Type	Material	Failure time	Volume (m^3)	Monitoring method	Data source	Reference
Mud Greek	USA	Rockslide	Shale, sandstone, sediments	2017-05-20	3×10^{6}	Satellite-based InSAR	Digitized	34
Nevis Bluff	New Zealand	Flexural topple / rockslide	Schist	1975-06-14	3.2×10^{4}	Survey markers	Digitized	35
New Tredegar	UK	Rockslide	Sandstone	1930-04-12	$\sim7\times10^{4}$	Unspecified	Digitized	36
Northern Bohemia	Czech Republic	Rockfall	Sandstone	1984-01-07	1.4×10^{3}	Extensometers	Digitized	37
Open pit mine (event 3)	Undisclosed	Rockslide	Anorthosite	2014-09-26	6×10^{2}	Ground-based InSAR	Digitized	38
Open pit mine (event 4)	Undisclosed	Rockslide	Anorthosite	2014-2017	3×10^{3}	Ground-based InSAR	Digitized	38
Open pit mine (event 5)	Undisclosed	Topple	Anorthosite	2017-02-05	4×10^{3}	Ground-based InSAR	Digitized	38
Otomura	Japan	Rockslide	Sandstone & shale	2004-08-10	2×10^{5}	Extensometers	Digitized	39
Preonzo	Switzerland	Rockslide	Gneiss	2012-05-15	2.1×10^{5}	Extensometers & total station with reflectors	Original	40
Puigcercós	Spain	Rockfall	Marl, silt, sandstone, limestone	2013-12-03	1×10^{3}	LiDAR	Digitized	41
Road slope (event 1)	Undisclosed	Rockslide	Mobilized gneiss	2009-01-24	3×10^{2}	Ground-based InSAR	Digitized	42
Road slope (event 2)	Undisclosed	Flow	Colluvium	2009-02-18	1.4×10^{1}	Ground-based InSAR	Digitized	42
Road slope (event 3)	Undisclosed	Soilslide	Colluvium & beton	2009-12-20	1.6×10^{2}	Ground-based InSAR	Digitized	42
Road slope (event 4)	Undisclosed	Soilslide	Colluvium & beton	2010-01-16	2×10^{2}	Ground-based InSAR	Digitized	42
Road slope (event 5)	Undisclosed	Soilslide	Colluvium & beton	2009-02-03	8×10^{1}	Ground-based InSAR	Digitized	42
Road slope (event 6)	Undisclosed	Soilslide	Colluvium & beton	2010-02-11	5×10^{2}	Ground-based InSAR	Digitized	42
Road slope (event 7)	Undisclosed	Flow	Mobilized & altered gneiss	2010-02-12	2×10^{2}	Ground-based InSAR	Digitized	42
Road slope (event 8)	Undisclosed	Rockslide	Colluvium & beton	2010-02-12	3×10^{2}	Ground-based InSAR	Digitized	42
Road slope (event 9)	Undisclosed	Rockslide	Mobilized & altered gneiss	2010-02-17	8×10^{1}	Ground-based InSAR	Digitized	42
Road slope (event 10)	Undisclosed	Rockslide	Mobilized & altered gneiss	2010-03-10	1.5×10^{2}	Ground-based InSAR	Digitized	42

Supplementary Table 1 (continued). Landslide cases (49 events in total).

Site	Location	Type	Material	Failure time	Volume (m^3)	Monitoring method	Data source	Reference
Roesgrenda	Norway	Soilslide	Quick clay	2000-03-02	2×10^3	Extensometers	Digitized	43
Takabayama	Japan	Rockslide	Mudstone, sandstone	1970-01-22	5×10^3	Extensometers	Digitized	44
Vajont	Italy	Rockslide	Limestone	1963-10-09	2.7×10^8	Geodetic bench marks	Digitized	45
Veslemannen	Norway	Rockslide	Gneiss	2019-09-05	5.4×10^4	Ground-based InSAR	Original	46
Welland	Canada	Soilslide	Clay	1967-02-22	5×10^2	Extensometers	Digitized	47
Xintan	China	Rockslide	Sediments	1985-06-12	3×10^7	Geodetic bench marks	Digitized	48
Yusuihsi	Taiwan	Rockslide	Slate & phyllite	2021-08-07	8.5×10^6	Optical satellite imagery	Digitized	49

Note: InSAR - Interferometric Synthetic Aperture Radar; LiDAR - Light Detection and Ranging; GNSS - Global navigation satellite system.

Supplementary Table 2. Rockburst cases (11 events in total).

Site	Location	Material	Failure time	Volume (m^3)	Monitoring method	Data source	Reference
Coal mine (cut-through #4)	Australia	Coal	2004-06-11	$\sim 5\times10^1$	Extensometer	Digitized	50
Coal mine (cut-through #5)	Australia	Coal	2004-06-04	$\sim 5\times10^1$	Extensometer	Digitized	50
Gold mine (event 1)	South Africa	Metamorphic rock	1997-02-05	$\sim 1\times10^4$	Seismic stations	Digitized	51
Gold mine (event 2)	South Africa	Metamorphic rock	1997-04-03	$\sim 5\times10^3$	Seismic stations	Digitized	51
Gold mine (event 3)	South Africa	Metamorphic rock	1997-04-06	$\sim 5\times10^4$	Seismic stations	Digitized	51
Gold mine (event 4)	South Africa	Metamorphic rock	1997-05-04	$\sim 3\times10^4$	Seismic stations	Digitized	51
Gold mine (event 5)	South Africa	Metamorphic rock	1997-05-26	$\sim 3\times10^3$	Seismic stations	Digitized	51
Gold mine (event 6)	South Africa	Metamorphic rock	1997-06-06	$\sim 1\times10^4$	Seismic stations	Digitized	51
Gold mine (event 7)	South Africa	Metamorphic rock	1997-06-23	$\sim 4\times10^3$	Seismic stations	Digitized	51
Gold mine (event 8)	South Africa	Metamorphic rock	1997-08-15	$\sim 4\times10^3$	Seismic stations	Digitized	51
Platinum mine	South Africa	Merensky reef	2007-04-04	$\sim 1\times10^1$	Closure meters	Digitized	52

Note: the volumes of the rockburst events at the Gold mine are estimated based on the documented seismic moment.

Supplementary Table 3. Glacier cases (17 events in total).

Site	Location	Type	Failure time	Volume (m^3)	Monitoring method	Data source	Reference
Amery	Antarctica	Ice shelf	2019-09-25	3.4×10^{11}	Satellite images	Digitized	53
Eiger glacier (2001 event)	Switzerland	Polythermal glacier	2001-08-20	2.7×10^{5}	Theodolite with reflectors	Digitized	54
Eiger glacier (2016 event)	Switzerland	Polythermal glacier	2016-08-25	1.5×10^{4}	Ground-based InSAR	Digitized	55
Grandes Jorasses (2014 event)	Italy	Cold glacier	2014-09-23 & 2014-09-29	1.5×10^{5}	Total station with reflectors	Original	56
Grandes Jorasses (2020 event)	Italy	Cold glacier	2020-11-11	2×10^{4}	Total station with reflectors	Digitized	57
Gruben	Switzerland	Temperate glacier	1974-09-09	8×10^{3}	Dial gauge with wire	Digitized	54
Mönch	Switzerland	Temperate glacier	2003-07-04	6×10^{5}	Theodolite with reflectors	Original	54
Planpincieux (event 1)	Italy	Polythermal glacier	2015-08-16	1.4×10^{4}	Time-lapse camera	Original	57,58
Planpincieux (event 2)	Italy	Polythermal glacier	2016-08-15	2.5×10^{4}	Time-lapse camera	Original	57,58
Planpincieux (event 3)	Italy	Polythermal glacier	2017-08-03	2×10^{4}	Time-lapse camera	Original	57,58
Planpincieux (event 4)	Italy	Polythermal glacier	2017-08-31	5.4×10^{4}	Time-lapse camera	Original	57,58
Planpincieux (event 5)	Italy	Polythermal glacier	2017-10-12	1.5×10^{4}	Time-lapse camera	Original	57,58
Planpincieux (event 6)	Italy	Polythermal glacier	2019-07-26	2.2×10^{4}	Time-lapse camera	Original	57,58
UK211	Antarctica	Iceberg	2006-11-23	2.4×10^{9}	Satellite images	Digitized	59
Weisshorn (1973 event)	Switzerland	Cold glacier	1972-10-17	5×10^{5}	Theodolite with reflectors	Original	60
Weisshorn (2005 event)	Switzerland	Cold glacier	2005-03-30	5×10^{5}	Total station with reflectors	Original	60,61
Weissmies	Switzerland	Polythermal glacier	2017-09-10	2.5×10^{5}	Time-lapse camera	Original	9,62

Supplementary Table 4. Volcano cases (32 events in total).

Site	Location	Type	Eruption time	Erupted volume (m^3)	Monitoring method	Data source	Reference
Adatara	Japan	Stratovolcano	1996-09-01	1.1×10^6	JMA seismic network	Original	10
Asama	Japan	Complex volcano	2009-02-02	$\sim10^4$	JMA seismic network	Original	10
Augustine	USA	Lava dome	2006-01-11	7.3×10^7	USGS seismic network-AVO	Original	10
Axial Seamount	Pacific Ocean	Submarine fissure volcano	2011-04-06	9.9×10^7	Bottom pressure recorders & mobile pressure recorders	Original	63
Bezymianny	Russia	Stratovolcano	1960-04-10	$\sim10^6$	Unspecified	Digitized	2
Etna (1989 event)	Italy	Stratovolcano	1989-09-08	$\sim10^7$	ISCSN	Original	10
Etna (2013 event)	Italy	Stratovolcano	2013-09-05	$\sim10^6$	Geochemical monitoring stations	Original	10,64
Hierro	Spain	Submarine shield volcano	2011-10-10	3.3×10^7	El Hierro seismic network	Original	10,65
Kilauea (1971 event)	USA	Shield volcano	1971-08-14	9.1×10^6	NCDC-ANSS seismic network	Original	10
Kilauea (1972 event)	USA	Shield volcano	1972-02-04	1.2×10^8	NCDC-ANSS seismic network	Original	10
Kilauea (1983 event)	USA	Shield volcano	1983-01-03	4×10^6	NCDC-ANSS seismic network	Original	10
Kujusan	Japan	Stratovolcano	1995-10-11	2×10^5	JMA seismic network	Original	10,66
Mauna Loa	USA	Shield volcano	1984-03-25	$\sim10^8$	Unspecified	Digitized	67
Merapi (2006 event)	Indonesia	Stratovolcano	2006-06-06	5.3×10^6	Plawangan Observatory	Original	10,68
Merapi (2010 event)	Indonesia	Stratovolcano	2010-10-26	$\sim10^7$	Plawangan Observatory	Original	10,69
Pinatubo	Philippines	Stratovolcano	1991-06-07	5×10^9	Seismic network	Original	10
Redoubt (1989 event)	USA	Stratovolcano	1989-12-14	$\sim10^8$	ANSS seismic network	Original	10,70
Redoubt (2009 event)	USA	Stratovolcano	2009-03-22	$\sim10^8$	ANSS seismic network	Original	10,71
Ruapehu (1995 event)	New Zealand	Stratovolcano	1995-09-18	2×10^5	New Zealand seismic network	Original	10
Ruapehu (1996 event)	New Zealand	Stratovolcano	1996-06-19	$\sim10^7$	New Zealand seismic network	Original	10
Ruapehu (2006 event)	New Zealand	Stratovolcano	2006-10-04	$\sim10^5$	New Zealand seismic network	Original	10
Sakurajima	Japan	Stratovolcano	2017-03-25	$\sim10^6$	JMA seismic network	Original	10

Supplementary Table 4 (continued). Volcano cases (32 events in total).

Site	Location	Type	Eruption time	Erupted volume (m^3)	Monitoring method	Data source	Reference
Sierra Negra (2005 event)	Ecuador	Shield volcano	2005-10-22	1.5×10^8	Continuous GPS network	Original	72
Sierra Negra (2018 event)	Ecuador	Shield volcano	2018-06-26	1.4×10^8	Continuous GPS network	Original	73
St. Helens (1980 event)	USA	Stratovolcano	1980-03-27	$\sim10^5$	PNSN	Original	10,74
St. Helens (1981 event)	USA	Stratovolcano	1981-09-06	$\sim10^6$	PNSN	Original	10,74
St. Helens (1982 event)	USA	Stratovolcano	1982-03-19	$\sim10^6$	PNSN, tiltmeter	Original	10,74
St. Helens (1985 event)	USA	Stratovolcano	1985-05-25	$\sim10^6$	PNSN	Original	10,75
Soufriere Hills	UK	Stratovolcano	1995-11-15	7×10^7	ISCSN	Original	10,76
Tokachidake	Japan	Stratovolcano	2004-02-25	$\sim10^5$	JMA seismic network	Original	10
Unzen	Japan	Complex volcano	1990-11-17	2.1×10^8	ISCSN	Original	10,77
Yakedake	Japan	Stratovolcano	1995-02-11	6×10^3	JMA seismic network	Original	10

Note: PNSN - Pacific Northwest Seismic Network; ISCSN - International Seismological Centre Seismographic Network; JMASN - Japan Meteorological Agency Seismic Network; NCDC - National Climatic Data Center; ANSS - Advanced National Seismic System; GPS - Global Positioning System; USGS - United States Geological Survey; AVO - Alaska Volcano Observatory.

Supplementary Table 5. Parameters of the LPPLS and PLS models fitted to landslide data (49 events and 94 time series).

Data	LPPLS							PLS			
	t_c	m	ω	ϕ	A	B	C	t_c	m	A	B
Abbotsford	1.40	-0.24	4.94	1.56	-2.60e+00	5.90e+00	8.03e-02	1.19	-0.20	-3.12e+00	6.24e+00
Achoma	-0.06	0.076	5.01	-1.21	2.59e+01	-1.79e+01	1.84e-01	2.49	-0.13	-1.16e+01	2.27e+01
Agoyama	0.00	-0.35	5.27	0.29	-1.87e+00	7.23e+00	7.87e-02	0.00	-0.36	-1.84e+00	7.20e+00
Arvigo	0.17	-0.063	5.04	-1.28	-5.37e-01	7.29e-01	2.74e-03	0.17	-0.07	-4.40e-01	6.34e-01
Baishi	0.02	0.19	5.73	0.93	5.68e+01	-1.67e+01	1.23e-01	0.02	0.22	5.36e+01	-1.36e+01
Baiyan	26.78	0.33	4.94	0.49	1.96e-01	-3.10e-02	2.86e-03	31.17	-0.29	-1.25e-01	6.70e-01
Brienz/Brinzauls (reflector 715)	3.11	-0.53	4.94	-0.03	-1.40e+00	4.76e+01	5.79e-01	1.57	-0.39	-3.41e+00	3.64e+01
Brienz/Brinzauls (reflector 719)	3.40	-0.63	4.94	-0.20	-1.11e+00	8.53e+01	1.23e+00	3.40	-0.64	-1.09e+00	8.62e+01
Brienz/Brinzauls (reflector 725)	3.22	-0.61	4.94	-0.10	-8.45e-01	6.89e+01	9.67e-01	1.62	-0.45	-2.79e+00	4.91e+01
Cadia	16.16	-0.8	4.94	-0.43	2.78e-03	1.09e+00	6.15e-02	19.48	-0.87	4.31e-03	1.44e+00
Copper open pit	10.28	-1.2	9.88	-0.16	1.82e-03	1.15e+00	1.88e-01	10.28	-1.28	2.30e-03	1.55e+00
Dosan (point 1)	0.06	-0.15	9.41	0.24	-7.73e-02	8.90e-02	8.68e-04	0.10	-0.27	-4.28e-02	5.50e-02
Dosan (point 2)	0.16	-0.52	4.94	-0.43	-5.63e-02	8.71e-02	2.06e-03	0.18	-0.57	-4.86e-02	8.07e-02
Dosan (point 3)	0.05	-0.38	4.94	0.94	-1.63e-01	2.55e-01	2.12e-03	0.05	-0.38	-1.59e-01	2.51e-01
Gallivaggio	0.85	-1.2	11.63	-1.46	3.54e-03	5.78e-03	4.16e-04	0.85	-1.20	3.56e-03	5.89e-03
Galterengraben (TJM1)	32.07	-0.49	5.99	-1.51	-4.97e+00	8.83e+01	3.47e+00	36.07	-0.42	-6.65e+00	7.95e+01
Galterengraben (TJM2)	30.83	-0.53	4.94	-0.19	-4.31e+00	1.29e+02	5.47e+00	34.67	-0.53	-4.49e+00	1.35e+02
Galterengraben (TJM6)	12.96	-0.51	4.94	-1.56	-1.22e-01	3.12e+01	1.38e+00	6.52	-0.31	-1.66e+00	1.85e+01
Grabengufer (GNSS)	0.24	-0.35	12.42	1.16	7.71e+00	4.28e-01	2.75e-02	-0.01	0.13	8.95e+00	-8.36e-01
Grabengufer (inclinometer W)	0.23	-1.2	4.94	-1.39	2.60e+00	1.44e+00	3.06e-01	0.03	-0.46	1.02e+00	2.77e+00
Grabengufer (inclinometer N)	0.03	0.1	6.70	-0.84	7.55e+01	-7.59e+01	5.56e-01	0.03	0.13	5.83e+01	-5.88e+01
Hogarth (extensometer 1)	3.11	-2.7	4.94	1.05	3.09e-03	8.73e+00	2.23e+00	0.00	-1.20	2.06e-03	1.07e-01
Hogarth (extensometer 2)	0.00	-1.3	9.86	-1.22	7.35e-03	1.48e-01	9.85e-03	0.00	-1.37	7.50e-03	1.81e-01
Hogarth (extensometer 3)	6.76	-1.8	14.45	-1.43	5.01e-03	7.06e-01	3.98e-02	0.00	-1.19	4.81e-03	8.05e-02
Hogarth (extensometer 4)	0.00	-0.87	5.39	-0.81	8.56e-03	4.85e-02	1.16e-03	0.00	-0.89	8.61e-03	5.12e-02
Hogarth (extensometer 6)	7.11	-1.6	6.98	-0.01	1.14e-02	4.47e-01	9.90e-03	5.33	-1.53	1.14e-02	2.83e-01
Iron mine	0.43	-0.13	11.46	0.21	-9.44e-01	1.64e+00	5.84e-03	0.43	-0.15	-8.46e-01	1.55e+00
Jinlonggou	4.78	-0.87	4.94	-1.36	-5.56e-03	2.19e-01	2.52e-02	-1.00	-0.22	-2.89e-02	7.09e-02

Supplementary Table 5 (continued). Parameters of the LPPLS and PLS models fitted to landslide data (49 events and 94 time series).

Data	LPPLS							PLS			
	t_c	m	ω	ϕ	A	B	C	t_c	m	A	B
Kagemori (point 1)	3.11	0.14	4.94	1.52	5.04e-01	-1.59e-01	7.03e-04	3.50	0.09	6.36e-01	-2.83e-01
Kagemori (point 3)	12.44	0.22	4.94	1.21	6.91e-01	-2.00e-01	3.32e-03	14.00	0.12	1.12e+00	-5.58e-01
Kagemori (point 13)	2.84	0.57	4.94	0.45	2.82e-01	-1.29e-02	2.17e-04	3.20	0.71	2.72e-01	-7.01e-03
Kagemori (point 15)	4.53	-0.45	4.94	0.63	1.48e-01	8.12e-01	2.42e-02	0.00	-0.10	-3.87e-01	9.67e-01
Kagemori (point 17)	3.73	0.012	6.19	1.31	5.86e+00	-5.44e+00	5.51e-04	4.20	-0.01	-8.55e+00	8.98e+00
Kagemori (point 18)	0.00	0.21	4.94	-0.95	6.95e-01	-2.05e-01	1.70e-03	0.00	0.24	6.49e-01	-1.63e-01
Kagemori (point 21)	4.44	-0.95	7.20	0.72	1.03e-02	1.07e+00	7.21e-02	5.00	-0.93	7.49e-03	1.11e+00
Kagemori (point 23)	2.89	-0.4	6.67	-0.21	-7.85e-02	8.65e-01	1.77e-02	4.33	-0.48	-5.52e-02	1.04e+00
La Saxe	5.64	-3.6	7.72	-0.46	3.47e+00	6.57e+03	3.09e+03	6.34	-2.43	3.39e+00	5.83e+02
Letlhakane diamond mine	9.53	-0.37	4.94	1.20	-4.44e-01	2.29e+00	1.32e-01	10.61	-0.50	-2.81e-01	2.61e+00
Longjing	2.22	-0.45	4.94	-1.24	-1.66e-01	1.08e+00	2.84e-02	0.95	-0.04	-4.19e+00	4.77e+00
Maoxian (point 1)	2.86	0.17	5.31	0.53	1.12e-01	-3.42e-02	8.37e-04	2.86	0.18	1.09e-01	-3.14e-02
Maoxian (point 2)	2.86	0.15	5.32	0.43	1.69e-01	-5.89e-02	6.38e-04	2.86	0.15	1.65e-01	-5.47e-02
Maoxian (point 3)	2.86	0.14	5.29	0.69	1.75e-01	-6.48e-02	8.57e-04	2.86	0.14	1.73e-01	-6.19e-02
Mt. Beni	-0.86	-0.33	5.21	0.15	-1.02e+00	6.91e+00	1.23e-01	1.73	-0.45	-6.44e-01	8.76e+00
Mud Greek	42.13	-0.46	4.94	0.32	-2.88e-01	5.01e+00	5.57e-01	47.38	-0.52	-1.98e-01	5.74e+00
Nevis Bluff (point 1)	-3.11	-0.11	4.94	1.11	-5.28e-01	9.42e-01	5.57e-03	-2.00	-0.21	-2.20e-01	7.06e-01
Nevis Bluff (point 2)	-4.80	-0.051	4.94	-0.95	-2.07e+00	2.71e+00	5.33e-03	-3.90	-0.13	-6.87e-01	1.41e+00
Nevis Bluff (point A)	-3.38	-0.077	4.94	1.42	-1.14e+00	1.75e+00	6.00e-03	-2.30	-0.16	-4.58e-01	1.16e+00
New Tredegar	1.63	-0.58	5.93	0.96	6.11e-02	1.35e+00	3.52e-02	1.21	-0.52	4.21e-02	1.21e+00
Northern Bohemia	3.94	-0.68	4.94	0.12	7.62e-03	6.59e-02	9.97e-03	4.35	-0.64	7.64e-03	5.98e-02
Open pit mine (event 3)	0.03	-1.4	10.94	-0.14	1.50e-02	2.64e-03	1.08e-04	0.04	-1.49	1.51e-02	2.49e-03
Open pit mine (event 4)	0.13	0.041	4.94	0.81	1.92e+00	-1.83e+00	1.27e-02	0.00	0.38	2.85e-01	-2.14e-01
Open pit mine (event 5)	0.25	-0.1	5.97	-0.94	-1.33e-01	1.67e-01	2.54e-03	0.25	0.13	1.63e-01	-1.29e-01
Otomura	0.53	-0.81	4.94	-1.07	4.17e-02	3.12e-01	1.67e-02	0.00	-0.65	3.48e-02	2.37e-01

Supplementary Table 5 (continued). Parameters of the LPPLS and PLS models fitted to landslide data (49 events and 94 time series).

Data	LPPLS							PLS			
	t_c	*m*	*ω*	*ϕ*	*A*	*B*	*C*	t_c	*m*	*A*	*B*
Preonzo (extensometer 1)	5.95	-1.1	6.47	1.15	5.44e-01	1.84e+00	1.11e-01	6.59	-1.14	5.42e-01	2.01e+00
Preonzo (extensometer 2)	3.62	-0.65	4.94	-0.15	5.54e-01	6.72e-01	3.24e-02	4.99	-0.85	5.74e-01	1.04e+00
Preonzo (extensometer 3)	5.51	-1.4	5.16	-1.44	6.19e-01	7.31e+00	4.50e-01	6.09	-1.35	6.15e-01	7.86e+00
Preonzo (extensometer 4)	5.42	-1.5	6.63	1.27	7.32e-01	1.21e+01	7.97e-01	4.85	-1.30	7.20e-01	7.89e+00
Preonzo (extensometer 5)	4.44	-1.3	5.97	0.20	5.39e-01	6.21e+00	5.58e-01	3.02	-1.01	5.19e-01	2.66e+00
Preonzo (reflector 2)	2.49	-0.93	4.94	0.77	3.45e-01	4.80e+00	4.18e-01	0.89	-0.50	1.21e-01	2.32e+00
Preonzo (reflector 4)	2.40	-0.87	4.94	0.89	3.69e-01	4.43e+00	3.30e-01	0.89	-0.47	1.18e-01	2.36e+00
Preonzo (reflector 5)	2.35	-0.79	4.94	0.93	2.56e-01	2.83e+00	1.97e-01	0.89	-0.43	5.77e-02	1.65e+00
Preonzo (reflector 8)	2.35	-0.76	4.94	0.94	1.67e-01	1.74e+00	1.14e-01	0.89	-0.41	2.97e-02	1.06e+00
Preonzo (reflector 9)	3.82	-1.1	5.61	1.47	2.24e-01	2.81e+00	2.12e-01	2.35	-0.79	1.98e-01	1.33e+00
Puigcercós (area 4)	191.03	0.63	4.94	-1.48	2.42e-01	-2.01e-03	8.77e-05	2.87	0.69	1.96e-01	-1.06e-03
Puigcercós (area 6)	151.36	-0.67	4.94	-0.52	-9.30e-02	1.35e+01	7.96e-01	73.42	-0.36	-1.92e-01	2.76e+00
Puigcercós (area 7)	189.80	-0.5	4.94	1.18	-1.16e-01	5.03e+00	4.20e-01	1.35	0.14	5.20e-01	-1.85e-01
Puigcercós (area 9)	130.55	-1.7	9.16	-1.44	-7.31e-03	9.23e+02	2.53e+02	33.62	-0.83	-1.81e-02	5.80e+00
Road slope (event 1)	0.13	-0.41	4.94	0.03	-6.02e-02	9.56e-02	2.58e-03	0.07	-0.25	-1.12e-01	1.45e-01
Road slope (event 2)	0.86	0.031	6.89	1.45	1.18e+01	-1.10e+01	2.80e-02	0.96	-0.05	-7.71e+00	8.54e+00
Road slope (event 3)	-0.05	0.0043	4.94	1.29	2.10e+00	-2.12e+00	6.94e-04	-0.05	-0.12	-6.23e-02	5.25e-02
Road slope (event 4)	0.01	-0.51	4.94	1.34	-1.75e-02	4.66e-03	4.90e-04	0.01	-0.51	-1.68e-02	4.72e-03
Road slope (event 5)	-0.03	-0.4	4.94	-0.92	-1.46e-02	9.38e-03	5.20e-04	-0.03	-0.54	-8.09e-03	4.92e-03
Road slope (event 6)	0.14	-0.46	4.94	-0.40	-2.37e-02	2.58e-02	1.28e-03	0.16	-0.58	-1.70e-02	1.94e-02
Road slope (event 7)	-0.03	-0.64	4.94	-0.11	1.12e-02	2.18e-02	2.06e-03	-0.10	-0.13	-1.13e-01	1.42e-01
Road slope (event 8)	0.02	0.19	4.94	0.42	2.27e-01	-2.83e-01	2.47e-03	0.02	0.09	4.13e-01	-4.56e-01
Road slope (event 9)	0.82	-0.92	6.87	0.53	-1.12e-01	9.80e-01	5.31e-02	0.93	-0.96	-1.11e-01	1.06e+00

Supplementary Table 5 (continued). Parameters of the LPPLS and PLS models fitted to landslide data (49 events and 94 time series).

Data	LPPLS							PLS			
	t_c	m	ω	ϕ	A	B	C	t_c	m	A	B
Road slope (event 10)	0.07	-0.75	4.94	0.64	-3.52e-02	2.58e-02	1.79e-03	0.07	-0.82	-2.87e-02	2.15e-02
Roesgrenda	-0.15	0.2	6.28	-1.23	1.05e-01	-3.84e-02	6.89e-04	-0.15	0.25	9.35e-02	-2.76e-02
Takabayama	-0.06	-0.28	4.94	-0.01	-9.23e-01	2.19e+00	2.84e-02	-0.06	-0.27	-9.75e-01	2.23e+00
Vajont (#2)	6.13	-0.98	5.59	0.33	2.98e+00	1.24e+01	1.30e+00	6.90	-0.89	2.90e+00	1.13e+01
Vajont (#4)	5.42	0.21	6.89	0.48	3.99e+00	-7.26e-01	5.85e-03	6.10	0.16	4.42e+00	-1.09e+00
Vajont (#6)	6.40	0.02	4.94	-0.59	2.76e+01	-2.28e+01	4.17e-02	7.20	-0.15	-1.05e-01	5.36e+00
Vajont (#58)	8.71	-0.025	4.94	0.52	-1.27e+01	1.68e+01	2.39e-02	9.80	-0.13	-3.72e-01	4.88e+00
Veslemannen (radar point 1)	0.13	0.65	6.52	-0.75	1.90e+01	-3.39e-01	3.06e-02	10.93	0.29	2.55e+01	-3.28e+00
Veslemannen (radar point 2)	0.13	0.42	7.49	-0.82	1.36e+01	-1.03e+00	4.09e-02	9.23	0.07	4.39e+01	-2.68e+01
Veslemannen (radar point 3)	0.13	0.37	6.46	-0.42	1.19e+01	-1.00e+00	4.16e-02	10.93	-0.04	-4.08e+01	5.68e+01
Veslemannen (radar point 4)	0.13	0.39	6.42	-0.27	6.61e+00	-5.03e-01	2.36e-02	9.64	0.03	4.06e+01	-3.22e+01
Veslemannen (radar point 5)	0.13	0.49	6.44	-0.31	5.70e+00	-2.13e-01	1.25e-02	10.53	0.15	9.63e+00	-2.87e+00
Veslemannen (radar point 6)	0.13	0.21	6.51	-0.48	4.54e+00	-1.15e+00	2.45e-02	1.26	0.11	7.00e+00	-3.35e+00
Veslemannen (radar point 7)	0.13	0.47	6.57	-0.81	4.23e+00	-2.03e-01	1.12e-02	10.23	0.14	7.85e+00	-2.74e+00
Welland (point 1)	0.28	-0.5	4.94	-1.41	-1.22e-03	1.37e-02	8.72e-04	0.31	-0.54	-3.01e-04	1.30e-02
Welland (point 2)	0.23	-0.47	4.96	-0.82	-1.02e-03	1.21e-02	9.11e-04	0.12	-0.31	-4.56e-03	1.50e-02
Xintan	12.89	-0.96	4.94	0.24	8.78e+00	7.04e+01	8.00e+00	15.36	-0.97	8.71e+00	8.45e+01
Yusuihsi	61.24	-0.66	4.94	-1.20	2.85e+00	2.17e+03	2.74e+02	65.70	-0.64	1.01e+00	2.10e+03

Note: t_c is in day; m, ω, and ϕ are dimensionless; A is in deg for tilt (Grabengufer inclinometer) and in meter for displacement (all other cases); B and C are in the unit of A per day^m. The actual failure corresponds to time t = 0 day. Parameters A, B, and C are displayed using the scientific exponential notation, where 1.00e-02 represents 1.00×10^{-2}.

Supplementary Table 6. Parameters of the LPPLS and PLS models fitted to rockburst data (11 events and 11 time series).

Data	LPPLS							PLS			
	t_c	m	ω	ϕ	A	B	C	t_c	m	A	B
Coal mine (cut-through #4)	0.12	-0.58	4.94	0.87	-7.14e+01	7.81e+01	3.86e+00	0.13	-0.62	-6.29e+01	7.15e+01
Coal mine (cut-through #5)	0.02	-0.12	4.94	-0.01	-2.43e-01	2.85e-01	5.77e-03	0.05	-0.28	-7.64e-02	1.19e-01
Gold mine (event 1)	-0.14	0.5	7.26	1.56	1.34e+03	-6.42e+02	4.43e+01	-0.14	0.43	1.40e+03	-7.14e+02
Gold mine (event 2)	0.08	-0.32	11.94	1.21	-4.91e+02	6.06e+02	3.61e+01	0.07	-0.09	-2.68e+03	2.80e+03
Gold mine (event 3)	1.01	0.24	9.59	-0.79	2.33e+04	-1.26e+04	2.11e+02	0.28	0.40	1.39e+04	-5.10e+03
Gold mine (event 4)	-2.46	0.59	9.14	0.36	1.36e+04	-1.34e+03	1.85e+01	-1.98	0.58	1.40e+04	-1.45e+03
Gold mine (event 5)	0.42	0.11	7.75	-1.40	2.31e+04	-1.70e+04	2.10e+02	0.97	0.01	2.12e+05	-2.05e+05
Gold mine (event 6)	0.03	0.42	8.37	1.04	3.28e+03	-1.51e+03	7.88e+01	0.03	0.44	3.22e+03	-1.45e+03
Gold mine (event 7)	3.75	0.43	4.98	-0.18	5.47e+03	-9.12e+02	3.47e+01	7.26	0.13	1.52e+04	-8.75e+03
Gold mine (event 8)	-5.42	0.64	5.65	0.26	4.14e+03	-2.12e+02	3.33e+01	4.74	0.09	2.06e+04	-1.33e+04
Platinum mine	0.04	0.31	4.94	1.38	2.35e-01	-3.56e-02	6.93e-04	0.04	0.36	2.31e-01	-3.07e-02

Note: t_c is in day; m, ω, and ϕ are dimensionless; A is in meter for displacement (Coal and Platinum mines) and in $kN^{1/2}km^{1/2}$ for Benioff strain (Gold mine); B and C are in the unit of A per day^m. The actual failure corresponds to time t = 0 day. Parameters A, B, and C are displayed using the scientific exponential notation, where 1.00e-02 represents 1.00×10^{-2}.

Supplementary Table 7. Parameters of the LPPLS and PLS models fitted to glacier data (17 events and 21 time series).

Data	LPPLS							PLS			
	t_c	m	ω	ϕ	A	B	C	t_c	m	A	B
Amery	2.69	0.32	10.33	-0.22	4.77e+01	-1.80e+00	4.33e-02	2.69	0.34	4.71e+01	-1.52e+00
Eiger glacier (2001 event)	-0.46	0.027	14.15	-0.06	5.10e+01	-4.83e+01	3.96e-03	-0.40	0.00	3.12e+02	-3.09e+02
Eiger glacier (2016 event)	0.00	0.51	4.94	-0.44	3.61e+00	-4.41e-01	3.43e-03	0.00	0.53	3.56e+00	-4.03e-01
Grandes Jorasses (2014 event, #13)	0.00	0.33	4.94	0.65	4.88e+01	-2.88e+00	3.56e-02	0.00	0.34	4.85e+01	-2.66e+00
Grandes Jorasses (2014 event, #14)	1.79	0.36	4.94	1.07	4.70e+01	-2.57e+00	6.86e-03	1.79	0.36	4.69e+01	-2.50e+00
Grandes Jorasses (2020 event)	0.26	0.36	5.80	0.62	2.34e+00	-9.28e-01	4.06e-03	0.26	0.35	2.36e+00	-9.52e-01
Gruben	0.03	0.5	8.11	0.16	2.14e+00	-1.20e+00	9.30e-03	0.03	0.50	2.14e+00	-1.20e+00
Mönch	-14.13	0.45	4.94	-1.10	7.27e+00	-1.04e+00	1.39e-02	-13.40	0.37	8.33e+00	-1.63e+00
Planpincieux (event 1)	0.00	0.58	4.94	0.41	1.85e+01	-1.38e+00	7.70e-03	0.00	0.60	1.83e+01	-1.31e+00
Planpincieux (event 2)	5.07	0.36	4.94	0.22	3.56e+01	-7.83e+00	8.69e-02	5.70	0.27	4.41e+01	-1.40e+01
Planpincieux (event 3)	0.00	0.53	4.94	1.19	2.26e+01	-2.42e+00	2.66e-02	1.73	0.43	2.67e+01	-4.34e+00
Planpincieux (event 4)	1.78	0.69	4.94	1.21	1.71e+01	-1.89e+00	6.52e-02	2.00	0.56	1.92e+01	-3.07e+00
Planpincieux (event 5)	0.00	0.7	7.41	1.28	1.13e+01	-1.00e+00	1.65e-02	0.00	0.72	1.11e+01	-9.04e-01
Planpincieux (event 6)	4.09	0.62	4.94	-0.50	1.36e+01	-1.15e+00	1.71e-02	4.60	0.62	1.39e+01	-1.16e+00
UK211	40.47	-0.36	4.94	-0.68	-1.12e+02	9.71e+02	4.90e+01	2.39	0.10	4.22e+02	-2.35e+02
Weisshorn (1973 event)	-9.84	0.21	4.94	-1.07	8.06e+01	-2.53e+01	2.40e-01	-7.03	0.11	1.42e+02	-7.66e+01
Weisshorn (2005 event, #103)	-0.92	0.56	4.94	-0.44	9.32e+00	-1.48e+00	7.17e-03	-1.18	0.57	9.11e+00	-1.44e+00
Weisshorn (2005 event, #104)	-0.92	0.53	4.94	-0.59	9.98e+00	-1.79e+00	6.59e-03	-0.92	0.52	1.01e+01	-1.86e+00
Weisshorn (2005 event, #105)	-0.92	0.44	4.94	-0.82	1.19e+01	-2.86e+00	1.11e-02	-0.66	0.41	1.27e+01	-3.37e+00
Weisshorn (2005 event, #106)	-2.99	0.36	9.24	1.19	1.28e+01	-4.11e+00	6.46e-03	-2.99	0.37	1.25e+01	-3.87e+00
Weissmies	0.75	0.39	11.20	1.09	8.52e+01	-8.41e+00	5.63e-02	0.75	0.40	8.48e+01	-8.12e+00

Note: t_c is in day; m, ω, and ϕ are dimensionless; A is in km^2 for area loss (UK211), in km for rift length (Amery), and in meter for displacement (all other cases); B and C are in the unit of A per day^m. The actual failure corresponds to time $t = 0$ day. Parameters A, B, and C are displayed using scientific exponential notation, where 1.00e-02 represents 1.00×10^{-2}.

Supplementary Table 8. Parameters of the LPPLS and PLS models fitted to volcano data (32 events and 34 time series).

Data	LPPLS							PLS			
	t_c	*m*	ω	ϕ	*A*	*B*	*C*	t_c	*m*	*A*	*B*
Adatara	93.18	0.36	7.12	0.90	2.20e+02	-1.22e+01	2.76e-01	105.70	0.29	2.49e+02	-2.33e+01
Asama	0.00	0.8	10.41	0.70	1.31e+04	-1.59e+02	6.40e+00	0.00	0.78	1.31e+04	-1.73e+02
Augustine	15.68	-0.31	4.96	-0.65	-4.19e+02	6.43e+03	1.69e+02	15.68	-0.28	-5.73e+02	6.12e+03
Axial Seamount	6.00	0.64	9.23	0.61	3.38e+00	-7.50e-03	5.12e-04	323.10	0.32	4.38e+00	-1.62e-01
Bezymianny	1.78	-1	4.94	0.41	-8.13e+00	2.93e+02	1.28e+01	2.11	-1.02	-8.36e+00	3.15e+02
Etna (1989 event)	31.62	-0.15	10.84	-0.91	-4.28e+02	1.10e+03	6.93e+00	35.20	-0.21	-3.02e+02	1.09e+03
Etna (2013 event)	0.35	0.76	4.94	-0.54	2.02e+03	-3.74e+01	8.23e-01	14.35	0.59	2.49e+03	-1.03e+02
Hierro	2.49	0.72	4.94	-0.41	1.20e+04	-4.56e+02	5.12e+01	11.20	0.38	2.03e+04	-3.40e+03
Kilauea (1971 event)	3.02	-0.12	4.94	0.33	-2.56e+03	4.33e+03	7.93e+01	0.00	0.30	1.64e+03	-4.80e+02
Kilauea (1972 event)	12.71	0.81	4.94	-0.48	8.03e+03	-5.86e+01	6.29e+00	43.20	0.55	1.06e+04	-3.46e+02
Kilauea (1983 event)	2.00	0.99	9.05	-0.29	6.58e+03	-1.56e+01	1.34e+00	34.20	0.82	7.46e+03	-4.50e+01
Kujusan	4.98	0.75	4.94	0.19	4.80e+01	-5.10e-01	7.93e-02	6.10	0.99	4.70e+01	-1.64e-01
Mauna Loa	5.73	-0.15	5.05	0.02	1.41e+01	7.75e+00	6.31e-02	4.30	-0.05	4.60e+00	1.65e+01
Merapi (2006 event)	5.51	0.39	4.94	-0.57	2.98e+04	-5.57e+03	8.98e+01	6.20	0.28	3.76e+04	-1.09e+04
Merapi (2010 event)	3.02	-0.35	5.44	-1.00	-3.90e+02	2.08e+03	2.74e+01	3.40	-0.41	-2.91e+02	2.14e+03
Pinatubo	0.36	-0.016	6.94	0.39	-2.90e+04	3.06e+04	6.06e+01	3.20	-0.50	-7.04e+02	4.50e+03
Redoubt (1989 event)	8.91	0.52	4.94	-0.16	7.26e+01	-3.40e+00	2.93e-01	3.00	0.79	6.10e+01	-6.93e-01
Redoubt (2009 event)	3.43	0.7	8.43	0.10	1.39e+03	-6.89e+01	3.93e+00	-1.00	0.72	1.24e+03	-5.90e+01
Ruapehu (1995 event)	13.60	0.67	6.40	0.32	3.87e+02	-7.26e+00	6.23e-01	0.00	0.99	3.36e+02	-1.26e+00
Ruapehu (1996 event)	9.07	0.83	4.94	0.75	1.84e+03	-1.79e+01	2.11e+00	10.20	0.99	1.83e+03	-9.04e+00
Ruapehu (2006 event)	25.07	0.45	11.66	1.50	9.22e+01	-6.70e+00	1.01e-01	28.20	0.40	1.02e+02	-1.02e+01
Sakurajima	8.18	-0.055	4.94	-0.88	-7.37e+03	9.66e+03	5.01e+01	9.20	-0.13	-2.67e+03	5.22e+03
Sierra Negra (2005 event)	85.89	-0.31	7.21	-0.92	-2.06e+00	1.63e+01	2.43e-01	96.00	-0.37	-1.69e+00	1.99e+01
Sierra Negra (2018 event)	154.60	-0.1	7.36	0.55	-8.32e+00	2.02e+01	4.79e-02	172.80	-0.15	-4.83e+00	1.92e+01
St. Helens (1980 event)	3.41	-3.4	7.42	-0.63	1.06e+01	1.64e+04	5.55e+03	0.13	-2.04	1.05e+01	2.51e+02

Supplementary Table 8 (continued). Parameters of the LPPLS and PLS models fitted to volcano data (32 events and 34 time series).

Data	LPPLS							PLS			
	t_c	*m*	*ω*	*ϕ*	*A*	*B*	*C*	t_c	*m*	*A*	*B*
St. Helens (1981 event)	0.20	0.73	4.94	0.14	9.98e+02	-1.42e+01	9.96e-01	0.20	0.86	9.76e+02	-7.42e+00
St. Helens (1982 event, seismic data)	4.62	-0.099	4.94	0.83	-1.97e+03	3.25e+03	4.32e+01	5.20	-0.31	-3.28e+02	1.92e+03
St. Helens (1982 event, radial tilt)	0.00	0.32	6.39	0.09	2.49e+03	-5.44e+02	9.76e+00	0.64	0.26	2.85e+03	-8.00e+02
St. Helens (1982 event, tangential tilt)	5.24	-0.4	6.14	0.25	-4.10e+03	6.24e+03	7.49e+01	5.90	-0.44	-4.00e+03	6.76e+03
St. Helens (1985 event)	3.29	-1.5	5.62	1.06	3.65e+01	2.88e+03	3.84e+02	3.70	-1.63	3.60e+01	3.86e+03
Soufriere Hills	-37.48	0.0039	5.50	0.60	2.92e+03	-2.79e+03	2.20e+00	-34.53	-0.04	-2.04e+02	3.38e+02
Tokachidake	4.98	0.99	9.38	0.95	2.73e+03	-1.26e+01	1.37e+00	22.40	0.80	3.18e+03	-3.78e+01
Unzen	0.00	0.82	6.11	-0.52	9.72e+01	-1.16e+00	1.49e-01	23.80	0.37	1.71e+02	-2.12e+01
Yakedake	6.20	0.99	4.94	-1.18	9.68e+01	-2.89e-01	2.44e-02	24.40	0.91	1.04e+02	-4.76e-01

Note: t_c is in day; *m*, *ω*, and *ϕ* are dimensionless; *A* is dimensionless for earthquake count, in meter for displacement, and in *μ*rad for tilt; *B* and C are in the unit of *A* per day^m. The actual failure corresponds to time *t* = 0 day. Parameters *A*, *B*, and *C* are displayed using scientific exponential notation, where 1.00e-02 represents 1.00×10^{-2}.

Supplementary Table 9. LPPLS versus PLS model comparison for landslides (49 events and 94 time series).

Data	LPPLS			PLS			*p*-value		
	NRMSE	NAIC	NBIC	NRMSE	NAIC	NBIC	Wilks	KS	AD
Abbotsford	4.98e-05	-6.01	-5.83	5.25e-04	-3.72	-3.61	0.00	0.00	0.00
Achoma	6.53e-03	0.19	0.51	9.20e-03	0.36	0.54	0.14	0.96	0.72
Agoyama	1.13e-04	-4.25	-3.97	1.97e-04	-3.84	-3.67	0.00	0.77	0.48
Arvigo	2.28e-05	-9.34	-9.10	3.53e-05	-9.00	-8.86	0.00	0.05	0.10
Baishi	2.36e-03	-0.01	0.31	3.51e-03	0.20	0.38	0.05	0.38	0.46
Baiyan	3.59e-04	-6.93	-6.60	1.16e-03	-5.96	-5.78	0.00	0.34	0.17
Brienz/Brinzauls (reflector 715)	3.56e-04	-2.01	-1.86	4.51e-04	-1.81	-1.73	0.00	0.04	0.17
Brienz/Brinzauls (reflector 719)	5.77e-04	-1.09	-0.95	8.25e-04	-0.77	-0.69	0.00	0.06	0.02
Brienz/Brinzauls (reflector 725)	4.80e-04	-1.43	-1.28	6.70e-04	-1.14	-1.06	0.00	0.12	0.17
Cadia	3.41e-05	-9.61	-9.27	4.75e-05	-9.54	-9.35	0.42	0.84	0.71
Copper open pit	6.52e-05	-8.94	-8.59	7.88e-05	-9.00	-8.80	0.52	0.99	0.73
Dosan (point 1)	9.47e-06	-11.45	-11.19	1.60e-05	-11.04	-10.89	0.00	0.18	0.14
Dosan (point 2)	1.57e-05	-9.91	-9.62	3.27e-05	-9.32	-9.16	0.00	0.03	0.09
Dosan (point 3)	9.96e-06	-9.15	-8.88	1.67e-05	-8.76	-8.61	0.00	0.21	0.27
Gallivaggio	1.12e-06	-15.61	-15.33	2.48e-06	-14.95	-14.79	0.00	0.61	0.60
Galterengraben (TJM1)	1.20e-03	-1.46	-1.38	4.50e-03	-0.15	-0.11	0.00	0.00	0.00
Galterengraben (TJM2)	2.26e-03	-0.53	-0.45	6.89e-03	0.57	0.61	0.00	0.01	0.00
Galterengraben (TJM6)	2.09e-03	-1.34	-1.26	3.32e-03	-0.90	-0.85	0.00	0.04	0.01
Grabengufer (GNSS)	8.41e-04	-4.82	-4.59	1.41e-03	-4.40	-4.26	0.00	0.69	0.68
Grabengufer (inclinometer W)	7.02e-03	0.49	0.75	8.07e-03	0.52	0.67	0.34	0.55	0.37
Grabengufer (inclinometer N)	2.16e-03	0.26	0.54	6.84e-03	1.27	1.43	0.00	0.27	0.08
Hogarth (extensometer 1)	3.50e-07	-16.66	-16.32	3.26e-06	-14.65	-14.46	0.00	0.28	0.17
Hogarth (extensometer 2)	1.29e-06	-15.11	-14.80	4.37e-06	-14.04	-13.87	0.00	0.12	0.21
Hogarth (extensometer 3)	2.24e-07	-17.61	-17.39	8.74e-07	-16.34	-16.21	0.00	0.15	0.08
Hogarth (extensometer 4)	2.66e-07	-17.20	-16.96	6.17e-07	-16.45	-16.31	0.00	0.00	0.00
Hogarth (extensometer 6)	1.31e-07	-18.23	-18.01	1.97e-07	-17.91	-17.78	0.00	0.18	0.18

Supplementary Table 9 (continued). LPPLS versus PLS model comparison for landslides (49 events and 94 time series).

Data	LPPLS			PLS			*p*-value		
	NRMSE	NAIC	NBIC	NRMSE	NAIC	NBIC	Wilks	KS	AD
Iron mine	1.73e-05	-8.35	-8.05	3.20e-05	-7.88	-7.71	0.00	0.98	0.67
Jinlonggou	8.70e-06	-11.79	-11.56	2.55e-05	-10.81	-10.68	0.00	0.09	0.01
Kagemori (point 1)	4.66e-06	-11.74	-11.43	1.08e-05	-11.07	-10.89	0.00	0.46	0.21
Kagemori (point 3)	5.33e-05	-8.30	-8.16	1.69e-04	-7.19	-7.11	0.00	0.00	0.00
Kagemori (point 13)	8.75e-07	-13.54	-13.21	5.30e-06	-11.96	-11.77	0.00	0.09	0.03
Kagemori (point 15)	2.88e-05	-8.93	-8.66	6.70e-05	-8.21	-8.05	0.00	0.09	0.02
Kagemori (point 17)	1.05e-06	-12.48	-12.19	1.58e-06	-12.21	-12.04	0.00	0.77	0.42
Kagemori (point 18)	2.84e-05	-8.78	-8.48	4.79e-05	-8.41	-8.24	0.00	0.04	0.06
Kagemori (point 21)	1.33e-05	-10.08	-9.81	7.73e-05	-8.44	-8.29	0.00	0.36	0.13
Kagemori (point 23)	7.97e-06	-10.03	-9.88	3.27e-05	-8.67	-8.58	0.00	0.00	0.00
La Saxe	1.95e-03	-1.79	-1.55	1.50e-02	0.16	0.29	0.00	0.12	0.07
Letlhakane diamond mine	2.51e-04	-5.91	-5.73	1.18e-03	-4.42	-4.32	0.00	0.00	0.00
Longjing	4.27e-05	-7.78	-7.57	1.24e-04	-6.80	-6.68	0.00	0.00	0.00
Maoxian (point 1)	8.45e-05	-8.91	-8.59	1.12e-04	-8.82	-8.64	0.35	0.94	0.76
Maoxian (point 2)	8.87e-05	-8.57	-8.24	9.80e-05	-8.66	-8.47	0.95	0.94	0.77
Maoxian (point 3)	8.80e-05	-8.57	-8.25	1.04e-04	-8.60	-8.41	0.74	0.94	0.75
Mt. Beni	1.25e-04	-4.81	-4.49	2.86e-04	-4.16	-3.98	0.00	0.23	0.24
Mud Greek	1.21e-03	-4.33	-4.03	3.25e-03	-3.50	-3.33	0.00	0.69	0.60
Nevis Bluff (point 1)	3.08e-05	-9.31	-9.13	6.23e-05	-8.67	-8.56	0.00	0.00	0.00
Nevis Bluff (point 2)	2.78e-05	-8.85	-8.64	5.22e-05	-8.29	-8.17	0.00	0.00	0.01
Nevis Bluff (point A)	2.48e-05	-9.10	-8.91	5.31e-05	-8.40	-8.29	0.00	0.00	0.00
New Tredegar	5.61e-05	-7.08	-6.78	9.85e-05	-6.67	-6.50	0.00	0.71	0.64
Northern Bohemia	1.78e-05	-11.95	-11.64	1.01e-04	-10.39	-10.21	0.00	0.18	0.05
Open pit mine (event 3)	7.27e-06	-10.44	-10.15	1.88e-05	-9.62	-9.46	0.00	0.92	0.75
Open pit mine (event 4)	1.60e-04	-7.23	-7.00	3.59e-04	-6.51	-6.38	0.00	0.06	0.02

Supplementary Table 9 (continued). LPPLS versus PLS model comparison for landslides (49 events and 94 time series).

Data	LPPLS			PLS			*p*-value		
	NRMSE	NAIC	NBIC	NRMSE	NAIC	NBIC	Wilks	KS	AD
Open pit mine (event 5)	1.71e-05	-11.21	-10.98	7.90e-05	-9.77	-9.64	0.00	0.01	0.00
Otomura	4.16e-05	-8.45	-8.18	4.76e-05	-8.44	-8.28	0.44	0.95	0.72
Preonzo (extensometer 1)	1.14e-05	-9.81	-9.56	3.90e-05	-8.69	-8.55	0.00	0.09	0.12
Preonzo (extensometer 2)	1.56e-05	-9.38	-9.09	7.09e-05	-8.01	-7.85	0.00	0.55	0.23
Preonzo (extensometer 3)	2.71e-05	-7.88	-7.62	8.45e-05	-6.86	-6.71	0.00	0.17	0.15
Preonzo (extensometer 4)	1.27e-05	-8.21	-7.94	1.10e-04	-6.16	-6.01	0.00	0.37	0.13
Preonzo (extensometer 5)	1.90e-05	-8.01	-7.78	1.29e-04	-6.19	-6.05	0.00	0.28	0.30
Preonzo (reflector 2)	1.09e-04	-5.24	-4.93	4.72e-04	-3.94	-3.76	0.00	0.03	0.06
Preonzo (reflector 4)	8.14e-05	-5.53	-5.21	4.54e-04	-3.98	-3.80	0.00	0.16	0.05
Preonzo (reflector 5)	6.35e-05	-6.15	-5.84	3.38e-04	-4.66	-4.48	0.00	0.08	0.05
Preonzo (reflector 8)	3.87e-05	-7.12	-6.80	2.09e-04	-5.62	-5.44	0.00	0.08	0.05
Preonzo (reflector 9)	2.22e-05	-8.15	-7.92	9.31e-05	-6.81	-6.68	0.00	0.30	0.29
Puigcercós (area 4)	1.53e-04	-6.82	-6.47	2.40e-04	-6.68	-6.48	0.35	0.46	0.54
Puigcercós (area 6)	6.97e-05	-6.95	-6.60	2.17e-04	-6.14	-5.94	0.03	0.43	0.16
Puigcercós (area 7)	1.11e-04	-6.84	-6.49	3.51e-04	-5.99	-5.79	0.00	0.13	0.06
Puigcercós (area 9)	9.27e-05	-7.15	-6.80	2.31e-04	-6.57	-6.37	0.05	0.71	0.35
Road slope (event 1)	1.11e-06	-12.62	-12.35	2.54e-05	-9.62	-9.46	0.00	0.00	0.00
Road slope (event 2)	2.01e-04	-5.91	-5.80	7.46e-04	-4.62	-4.56	0.00	0.00	0.00
Road slope (event 3)	4.88e-06	-13.13	-12.86	1.41e-05	-12.19	-12.04	0.00	0.02	0.01
Road slope (event 4)	1.82e-05	-11.60	-11.33	1.77e-04	-9.45	-9.30	0.00	0.00	0.00
Road slope (event 5)	2.49e-05	-11.51	-11.30	5.67e-05	-10.77	-10.65	0.00	0.00	0.00

Supplementary Table 9 (continued). LPPLS versus PLS model comparison for landslides (49 events and 94 time series).

Data	LPPLS			PLS			*p*-value		
	NRMSE	NAIC	NBIC	NRMSE	NAIC	NBIC	Wilks	KS	AD
Road slope (event 6)	2.07e-05	-11.14	-10.93	4.87e-05	-10.37	-10.25	0.00	0.01	0.00
Road slope (event 7)	5.26e-05	-9.05	-8.75	7.54e-05	-8.84	-8.67	0.02	0.89	0.55
Road slope (event 8)	1.01e-05	-11.11	-10.86	2.17e-05	-10.44	-10.30	0.00	0.48	0.18
Road slope (event 9)	1.15e-04	-6.29	-6.19	2.50e-04	-5.54	-5.48	0.00	0.00	0.00
Road slope (event 10)	4.65e-05	-8.93	-8.75	1.16e-04	-8.08	-7.97	0.00	0.00	0.00
Roesgrenda	3.24e-05	-10.34	-10.13	4.42e-05	-10.11	-9.99	0.00	0.10	0.12
Takabayama	9.01e-06	-8.11	-7.84	5.63e-05	-6.41	-6.25	0.00	0.00	0.00
Vajont (bench mark 2)	6.85e-04	-3.93	-3.70	1.70e-03	-3.11	-2.98	0.00	0.00	0.01
Vajont (bench mark 4)	3.78e-05	-7.61	-7.37	1.42e-04	-6.38	-6.24	0.00	0.04	0.00
Vajont (bench mark 6)	4.61e-04	-4.68	-4.46	1.18e-03	-3.82	-3.69	0.00	0.00	0.00
Vajont (bench mark 58)	9.32e-05	-6.42	-6.23	2.95e-04	-5.32	-5.22	0.00	0.00	0.00
Veslemannen (radar point 1)	1.14e-03	-2.02	-1.85	1.29e-02	0.34	0.44	0.00	0.00	0.00
Veslemannen (radar point 2)	5.55e-04	-2.84	-2.64	3.73e-03	-1.00	-0.89	0.00	0.00	0.00
Veslemannen (radar point 3)	4.34e-04	-3.28	-3.11	3.57e-03	-1.23	-1.13	0.00	0.00	0.00
Veslemannen (radar point 4)	3.42e-04	-4.08	-3.91	2.53e-03	-2.14	-2.04	0.00	0.00	0.00
Veslemannen (radar point 5)	2.69e-04	-4.69	-4.51	2.10e-03	-2.68	-2.58	0.00	0.00	0.00
Veslemannen (radar point 6)	1.93e-04	-5.00	-4.82	9.47e-04	-3.47	-3.37	0.00	0.00	0.00
Veslemannen (radar point 7)	2.51e-04	-4.93	-4.75	1.71e-03	-3.07	-2.96	0.00	0.00	0.00
Welland (point 1)	2.46e-06	-14.03	-13.80	1.91e-05	-12.06	-11.93	0.00	0.00	0.00
Welland (point 2)	6.32e-06	-13.08	-12.91	1.95e-05	-12.00	-11.91	0.00	0.04	0.00
Xintan	3.44e-03	-1.11	-0.78	6.74e-03	-0.63	-0.45	0.00	0.36	0.21
Yusuihsi	1.93e-02	3.42	3.58	2.33e-01	5.86	5.95	0.00	0.00	0.00

Note: NRMSE is displayed using scientific exponential notation, where 1.00e-02 represents 1.00×10^{-2}.

Supplementary Table 10. LPPLS versus PLS model comparison for rockbursts (11 events and 11 time series).

Data	LPPLS			PLS			*p*-value		
	NRMSE	NAIC	NBIC	NRMSE	NAIC	NBIC	Wilks	KS	AD
Coal mine (cut-through #4)	1.09e-01	5.86	6.10	1.89e-01	6.32	6.45	0.00	0.67	0.42
Coal mine (cut-through #5)	4.34e-05	-8.85	-8.61	1.42e-04	-7.76	-7.62	0.00	0.03	0.00
Gold mine (event 1)	4.60e-01	9.82	10.16	1.42e+00	10.66	10.86	0.00	0.53	0.33
Gold mine (event 2)	1.44e+00	10.89	11.21	2.02e+00	10.80	10.99	0.86	0.86	0.70
Gold mine (event 3)	4.71e+00	13.78	13.95	7.84e+00	14.23	14.33	0.00	0.09	0.03
Gold mine (event 4)	2.46e+00	13.00	13.16	3.32e+00	13.25	13.34	0.00	0.39	0.31
Gold mine (event 5)	3.52e+00	13.10	13.32	7.25e+00	13.75	13.87	0.00	0.20	0.07
Gold mine (event 6)	2.11e+00	11.91	12.21	3.19e+00	12.17	12.34	0.01	0.71	0.46
Gold mine (event 7)	3.68e+00	12.67	12.95	5.29e+00	12.90	13.06	0.01	0.94	0.73
Gold mine (event 8)	1.08e+01	13.86	14.20	2.63e+01	14.49	14.69	0.01	0.20	0.10
Platinum mine	9.62e-06	-11.37	-11.26	1.58e-05	-10.91	-10.84	0.00	0.00	0.00

Note: NRMSE is displayed using scientific exponential notation, where 1.00e-02 represents 1.00×10^{-2}.

Supplementary Table 11. LPPLS versus PLS model comparison for glaciers (17 events and 21 time series).

Data	LPPLS			PLS			*p*-value		
	NRMSE	NAIC	NBIC	NRMSE	NAIC	NBIC	Wilks	KS	AD
Amery	6.90e-03	1.00	1.13	1.11e-02	1.44	1.51	0.00	0.04	0.01
Eiger glacier (2001 event)	1.71e-05	-7.23	-7.00	2.19e-05	-7.08	-6.95	0.00	0.82	0.67
Eiger glacier (2016 event)	9.85e-05	-5.09	-4.80	1.29e-04	-4.97	-4.80	0.05	0.36	0.32
Grandes Jorasses (2014 event, prism 13)	2.56e-04	-2.80	-2.68	1.01e-03	-1.46	-1.39	0.00	0.00	0.00
Grandes Jorasses (2014 event, prism 14)	7.37e-05	-4.11	-3.98	1.10e-04	-3.75	-3.67	0.00	0.00	0.00
Grandes Jorasses (2020 event)	5.02e-06	-9.00	-8.88	1.62e-05	-7.86	-7.79	0.00	0.00	0.00
Gruben	2.00e-05	-6.17	-5.89	3.34e-05	-6.16	-5.99	0.71	0.43	0.46
Mönch	2.57e-04	-3.73	-3.50	6.46e-04	-2.90	-2.77	0.00	0.00	0.00
Planpincieux (event 1)	4.96e-05	-4.17	-3.94	1.17e-04	-3.41	-3.28	0.00	0.31	0.14
Planpincieux (event 2)	8.06e-04	-1.14	-0.89	2.28e-03	-0.20	-0.06	0.00	0.02	0.00
Planpincieux (event 3)	1.43e-04	-2.90	-2.64	4.75e-04	-1.81	-1.66	0.00	0.11	0.01
Planpincieux (event 4)	2.43e-04	-2.36	-2.01	4.23e-03	0.22	0.41	0.00	0.07	0.01
Planpincieux (event 5)	1.97e-04	-2.92	-2.60	8.03e-04	-1.71	-1.53	0.00	0.06	0.04
Planpincieux (event 6)	1.00e-04	-3.76	-3.49	6.15e-04	-2.08	-1.92	0.00	0.04	0.00
UK211	1.95e-01	6.62	6.94	3.68e-01	7.05	7.24	0.01	0.76	0.40
Weisshorn (1973 event)	5.31e-03	1.38	1.71	1.04e-02	1.86	2.04	0.00	0.12	0.08
Weisshorn (2005 event, #103)	1.46e-05	-6.17	-5.92	5.92e-05	-4.88	-4.74	0.00	0.00	0.00
Weisshorn (2005 event, #104)	1.10e-05	-6.44	-6.19	3.54e-05	-5.37	-5.23	0.00	0.06	0.01
Weisshorn (2005 event, #105)	2.45e-05	-5.58	-5.34	6.35e-05	-4.73	-4.59	0.00	0.04	0.01
Weisshorn (2005 event, #106)	1.37e-05	-6.36	-6.11	3.29e-05	-5.59	-5.44	0.00	0.08	0.04
Weissmies	1.93e-03	0.40	0.62	2.60e-03	0.62	0.75	0.00	0.28	0.27

Note: NRMSE is displayed using scientific exponential notation, where 1.00e-02 represents 1.00×10^{-2}.

Supplementary Table 12. LPPLS versus PLS model comparison for volcanoes (32 events and 34 time series).

Data	LPPLS			PLS			*p*-value		
	NRMSE	NAIC	NBIC	NRMSE	NAIC	NBIC	Wilks	KS	AD
Adatara	1.37e-02	3.11	3.24	5.67e-02	4.49	4.57	0.00	0.00	0.00
Asama	1.28e+00	12.53	12.63	5.09e+00	13.88	13.94	0.00	0.00	0.00
Augustine	4.00e-01	9.49	9.62	5.95e-01	9.85	9.93	0.00	0.01	0.07
Axial Seamount	6.02e-04	-4.31	-4.24	1.55e-03	-3.37	-3.34	0.00	0.00	0.00
Bezymianny	5.88e-03	2.64	2.99	1.47e-02	3.25	3.45	0.03	0.28	0.25
Etna (1989 event)	2.49e-02	4.67	4.95	4.89e-02	5.22	5.38	0.00	0.81	0.44
Etna (2013 event)	8.84e-03	5.55	5.69	7.78e-02	7.68	7.76	0.00	0.00	0.00
Hierro	4.33e+00	13.69	13.86	3.50e+01	15.73	15.82	0.00	0.00	0.00
Kilauea (1971 event)	3.14e-01	8.84	9.07	1.11e+00	10.02	10.15	0.00	0.02	0.01
Kilauea (1972 event)	9.35e-01	11.77	12.02	1.44e+01	14.41	14.55	0.00	0.00	0.00
Kilauea (1983 event)	2.03e+00	12.19	12.47	8.29e+00	13.47	13.63	0.00	0.01	0.01
Kujusan	2.75e-02	2.27	2.45	8.43e-02	3.33	3.44	0.00	0.05	0.00
Mauna Loa	2.90e-04	-4.42	-4.27	5.01e-04	-3.92	-3.83	0.00	0.04	0.02
Merapi (2006 event)	2.75e+00	13.73	13.97	5.48e+00	14.32	14.46	0.00	0.03	0.03
Merapi (2010 event)	8.49e-02	7.29	7.60	1.44e-01	7.65	7.82	0.01	0.64	0.47
Pinatubo	2.08e-01	8.99	9.31	9.89e-01	10.37	10.55	0.00	0.40	0.11
Redoubt (1989 event)	3.82e-02	3.96	4.26	1.07e-01	4.84	5.01	0.00	0.22	0.07
Redoubt (2009 event)	6.27e-01	9.70	9.95	1.31e+00	10.34	10.48	0.00	0.03	0.01
Ruapehu (1995 event)	8.01e-02	5.72	5.86	3.76e-01	7.23	7.30	0.00	0.00	0.00
Ruapehu (1996 event)	4.78e-01	9.12	9.30	1.67e+00	10.31	10.42	0.00	0.00	0.00
Ruapehu (2006 event)	2.47e-02	3.27	3.46	3.35e-02	3.52	3.62	0.00	0.87	0.58
Sakurajima	5.03e-01	9.11	9.30	1.17e+00	9.88	9.99	0.00	0.30	0.15
Sierra Negra (2005 event)	7.07e-04	-3.59	-3.35	1.03e-03	-3.31	-3.17	0.00	0.20	0.23
Sierra Negra (2018 event)	3.04e-04	-4.21	-4.05	3.92e-04	-4.01	-3.92	0.00	0.04	0.13
St. Helens (1980 event)	1.75e-03	1.22	1.46	6.85e-03	2.49	2.63	0.00	0.48	0.32

Supplementary Table 12 (continued). LPPLS versus PLS model comparison for volcanoes (32 events and 34 time series).

Data	LPPLS			PLS			*p*-value		
	NRMSE	NAIC	NBIC	NRMSE	NAIC	NBIC	Wilks	KS	AD
St. Helens (1981 event)	8.66e-02	6.81	6.96	2.97e-01	8.00	8.08	0.00	0.01	0.00
St. Helens (1982 event, seismic data)	4.27e-01	8.40	8.66	1.24e+00	9.35	9.50	0.00	0.01	0.01
St. Helens (1982 event, radial tilt)	1.32e-01	8.29	8.54	3.77e-01	9.24	9.38	0.00	0.15	0.06
St. Helens (1982 event, tangential tilt)	6.55e-02	7.82	8.06	1.66e-01	8.65	8.79	0.00	0.16	0.10
St. Helens (1985 event)	2.77e-02	5.28	5.58	1.53e-01	6.83	7.00	0.00	0.69	0.53
Soufriere Hills	4.00e-02	3.45	3.78	9.03e-02	4.07	4.25	0.00	0.54	0.43
Tokachidake	2.49e+00	11.84	12.16	9.57e+00	13.01	13.19	0.00	0.14	0.04
Unzen	5.30e-02	4.69	5.01	3.41e-01	6.39	6.56	0.00	0.05	0.01
Yakedake	3.52e-02	3.69	3.90	1.01e-01	4.67	4.79	0.00	0.04	0.01

Note: NRMSE is displayed using the scientific exponential notation, where 1.00e-02 represents 1.00×10^{-2}.

Supplementary Table 13. Parameters of the LPPLS and PLS calibration to landslide data (49 events and 94 time series).

	Calibration window size (day)	Number of data points	Aggregation interval (day)
Abbotsford	19.04	97	N/A
Achoma	114.76	34	N/A
Agoyama	42.00	43	1
Arvigo	63.00	63	1
Baishi	31.00	32	1
Baiyan	394.75	30	N/A
Brienz/Brinzauls (reflector 715)	139.00	140	1
Brienz/Brinzauls (reflector 719)	152.00	153	1
Brienz/Brinzauls (reflector 725)	144.00	145	1
Cadia	298.86	23	N/A
Copper open pit	241.73	24	N/A
Dosan (point 1)	2.17	53	0.042
Dosan (point 2)	1.75	43	0.042
Dosan (point 3)	1.92	47	0.042
Gallivaggio	26.18	45	1
Galterengraben (TJM1)	360.00	361	1
Galterengraben (TJM2)	346.00	347	1
Galterengraben (TJM6)	290.00	291	1
Grabengufer (GNSS)	2.83	66	0.042
Grabengufer (inclinometer W)	2.21	53	0.042
Grabengufer (inclinometer N)	1.75	43	0.042
Hogarth (extensometer 1)	35.00	27	1
Hogarth (extensometer 2)	45.00	38	1
Hogarth (extensometer 3)	76.00	68	1
Hogarth (extensometer 4)	72.00	65	1
Hogarth (extensometer 6)	80.00	73	1
Iron mine	39.00	40	1
Jinlonggou	65.00	66	1
Kagemori (point 1)	35.00	36	1
Kagemori (point 3)	140.00	141	1
Kagemori (point 13)	32.00	28	1
Kagemori (point 15)	51.00	49	1
Kagemori (point 17)	42.00	43	1
Kagemori (point 18)	39.00	39	1
Kagemori (point 21)	50.00	50	1
Kagemori (point 23)	130.00	129	1
La Saxe	63.00	63	1
Letlhakane diamond mine	97.22	105	N/A

Supplementary Table 13 (continued). Parameters of the LPPLS and PLS calibration to landslide data (49 events and 94 time series).

	Calibration window size (day)	Number of data points	Aggregation interval (day)
Longjing	14.29	75	N/A
Maoxian (point 1)	656.50	31	N/A
Maoxian (point 2)	656.50	31	N/A
Maoxian (point 3)	656.50	31	N/A
Mt. Beni	233.23	32	N/A
Mud Greek	472.80	38	N/A
Nevis Bluff (point 1)	100.00	101	1
Nevis Bluff (point 2)	81.00	82	1
Nevis Bluff (point A)	97.00	98	1
New Tredegar	37.94	39	N/A
Northern Bohemia	36.58	36	N/A
Open pit mine (event 3)	0.46	44	0.01
Open pit mine (event 4)	1.40	68	0.021
Open pit mine (event 5)	2.79	67	0.042
Otomura	48.00	49	1
Preonzo (extensometer 1)	57.00	57	1
Preonzo (extensometer 2)	41.00	41	1
Preonzo (extensometer 3)	52.00	52	1
Preonzo (extensometer 4)	51.00	51	1
Preonzo (extensometer 5)	64.00	65	1
Preonzo (reflector 2)	36.00	36	1
Preonzo (reflector 4)	34.00	34	1
Preonzo (reflector 5)	33.00	33	1
Preonzo (reflector 8)	33.00	33	1
Preonzo (reflector 9)	66.00	67	1
Puigcercós (area 4)	2116.87	19	N/A
Puigcercós (area 6)	1753.69	18	N/A
Puigcercós (area 7)	2120.11	20	N/A
Puigcercós (area 9)	1744.84	18	N/A
Road slope (event 1)	1.96	48	0.042
Road slope (event 2)	9.33	225	0.042
Road slope (event 3)	0.17	49	0.0035
Road slope (event 4)	0.08	48	0.0017
Road slope (event 5)	0.26	76	0.0035
Road slope (event 6)	1.58	76	0.021
Road slope (event 7)	0.83	40	0.021
Road slope (event 8)	0.21	60	0.0035
Road slope (event 9)	9.92	238	0.042
Road slope (event 10)	0.68	99	0.0069

Supplementary Table 13 (continued). Parameters of the LPPLS and PLS calibration to landslide data (49 events and 94 time series).

	Calibration window size (day)	Number of data points	Aggregation interval (day)
Roesgrenda	35.55	79	N/A
Takabayama	46.00	47	1
Vajont (bench mark 2)	69.00	70	1
Vajont (bench mark 4)	61.00	62	1
Vajont (bench mark 6)	72.00	73	1
Vajont (bench mark 58)	98.00	99	1
Veslemannen (radar point 1)	108.00	108	1
Veslemannen (radar point 2)	91.00	91	1
Veslemannen (radar point 3)	108.00	108	1
Veslemannen (radar point 4)	107.00	107	1
Veslemannen (radar point 5)	104.00	104	1
Veslemannen (radar point 6)	102.00	102	1
Veslemannen (radar point 7)	101.00	101	1
Welland (point 1)	2.96	71	0.042
Welland (point 2)	4.83	116	0.042
Xintan	221.87	31	7
Yusuihsi	847.00	122	7

Note: if no aggregation treatment is applied to the data, the aggregation interval is indicated as N/A.

Supplementary Table 14. Parameters of the LPPLS and PLS calibration to rockburst data (11 events and 11 time series).

	Calibration window size (day)	Number of data points	Aggregation interval (day)
Coal mine (cut-through #4)	1.33	64	0.021
Coal mine (cut-through #5)	2.58	63	0.042
Gold mine (event 1)	4.41	21	N/A
Gold mine (event 2)	1.34	14	N/A
Gold mine (event 3)	11.04	108	N/A
Gold mine (event 4)	43.01	125	N/A
Gold mine (event 5)	12.41	77	N/A
Gold mine (event 6)	5.77	39	N/A
Gold mine (event 7)	63.18	46	N/A
Gold mine (event 8)	114.20	23	N/A
Platinum mine	8.08	195	0.042

Note: if no aggregation treatment is applied to the data, the aggregation interval is indicated as N/A.

Supplementary Table 15. Parameters of the LPPLS and PLS calibration to glacier data (17 events and 21 time series).

	Calibration window size (day)	Number of data points	Aggregation interval (day)
Amery	2478.00	178	14
Eiger glacier (2001 event)	5.15	64	N/A
Eiger glacier (2016 event)	39.00	40	N/A
Grandes Jorasses (2014 event, prism 13)	178.00	179	1
Grandes Jorasses (2014 event, prism 14)	161.00	162	1
Grandes Jorasses (2020 event)	7.75	187	N/A
Gruben	3.21	12	N/A
Mönch	66.00	67	1
Planpincieux (event 1)	67.00	68	1
Planpincieux (event 2)	57.00	58	1
Planpincieux (event 3)	52.00	53	1
Planpincieux (event 4)	20.00	21	1
Planpincieux (event 5)	30.00	31	1
Planpincieux (event 6)	46.00	47	1
UK211	428.43	30	N/A
Weisshorn (1973 event)	252.70	31	7
Weisshorn (2005 event, #103)	23.26	54	N/A
Weisshorn (2005 event, #104)	23.26	59	N/A
Weisshorn (2005 event, #105)	23.26	62	N/A
Weisshorn (2005 event, #106)	23.26	56	N/A
Weissmies	75.00	76	1

Note: if no aggregation treatment is applied to the data, the aggregation interval is indicated as N/A.

Supplementary Table 16. Parameters of the LPPLS and PLS calibration to volcano data (32 events and 34 time series).

	Calibration window size (day)	Number of data points	Aggregation interval (day)
Adatara	1127.00	162	7
Asama	238.00	239	1
Augustine	1141.00	164	7
Axial Seamount	3171.00	454	7
Bezymianny	29.72	20	N/A
Etna (1989 event)	322.00	47	7
Etna (2013 event)	140.00	141	1
Hierro	112.00	113	1
Kilauea (1971 event)	34.00	69	0.5
Kilauea (1972 event)	392.00	57	7
Kilauea (1983 event)	322.00	47	7
Kujusan	101.00	102	1
Mauna Loa	129.00	130	1
Merapi (2006 event)	62.00	63	1
Merapi (2010 event)	34.00	35	1
Pinatubo	32.00	33	1
Redoubt (1989 event)	266.00	39	7
Redoubt (2009 event)	57.00	58	1
Ruapehu (1995 event)	153.00	154	1
Ruapehu (1996 event)	102.00	103	1
Ruapehu (2006 event)	282.00	95	3
Sakurajima	92.00	93	1
Sierra Negra (2005 event)	910.00	66	14
Sierra Negra (2018 event)	1638.00	118	14
St. Helens (1980 event)	59.00	60	1
St. Helens (1981 event)	142.00	143	1
St. Helens (1982 event, seismic data)	52.00	53	1
St. Helens (1982 event, radial tilt)	58.00	59	1
St. Helens (1982 event, tangential tilt)	59.00	60	1
St. Helens (1985 event)	37.00	38	1
Soufriere Hills	265.86	30	1
Tokachidake	224.00	33	7
Unzen	238.00	35	7
Yakedake	234.00	79	3

Note: if no aggregation treatment is applied to the data, the aggregation interval is indicated as N/A.

Supplementary References

1. Voight, B. A relation to describe rate-dependent material failure. *Science* **243**, 200–203 (1989).
2. Voight, B. A method for prediction of volcanic eruptions. *Nature* **332**, 125–130 (1988).
3. Sammis, C. G. & Sornette, D. Positive feedback, memory, and the predictability of earthquakes. *Proc. Natl. Acad. Sci.* **99**, 2501–2508 (2002).
4. Lei, Q. & Sornette, D. A stochastic dynamical model of slope creep and failure. *Geophys. Res. Lett.* **50**, e2022GL102587 (2023).
5. Sornette, D. *Critical Phenomena in Natural Sciences - Chaos, Fractals, Selforganization and Disorder: Concepts and Tools*. (Springer, Berlin/Heidelberg, 2006). doi:10.1007/3-540-33182-4.
6. Sornette, D. Discrete-scale invariance and complex dimensions. *Phys. Rep.* **297**, 239–270 (1998).
7. Gluzman, S. & Sornette, D. Log-periodic route to fractal functions. *Phys. Rev. E* **65**, 036142 (2002).
8. Sornette, D. & Sammis, C. G. Complex critical exponents from renormalization group theory of earthquakes: Implications for earthquake predictions. *J. Phys. I* **5**, 607–619 (1995).
9. Leinauer, J., Weber, S., Cicoira, A., Beutel, J. & Krautblatter, M. An approach for prospective forecasting of rock slope failure time. *Commun. Earth Environ.* **4**, 253 (2023).
10. Newhall, C. G. *et al.* WOVOdat – An online, growing library of worldwide volcanic unrest. *J. Volcanol. Geotherm. Res.* **345**, 184–199 (2017).
11. Filimonov, V. & Sornette, D. A stable and robust calibration scheme of the log-periodic power law model. *Phys. Stat. Mech. Its Appl.* **392**, 3698–3707 (2013).
12. Saleur, H., Sammis, C. G. & Sornette, D. Renormalization group theory of earthquakes. *Nonlinear Process. Geophys.* **3**, 102–109 (1996).
13. Saleur, H. & Sornette, D. Complex exponents and log-periodic corrections in frustrated systems. *J. Phys. I* **6**, 327–355 (1996).
14. Demos, G. & Sornette, D. Comparing nested data sets and objectively determining financial bubbles' inceptions. *Phys. Stat. Mech. Its Appl.* **524**, 661–675 (2019).
15. Hancox, G. T. The 1979 Abbotsford Landslide, Dunedin, New Zealand: a retrospective look at its nature and causes. *Landslides* **5**, 177–188 (2008).

16. Lacroix, P., Huanca, J., Albinez, L. & Taipe, E. Precursory motion and time-of-failure prediction of the Achoma landslide, Peru, from high frequency PlanetScope satellites. *Geophys. Res. Lett.* **50**, e2023GL105413 (2023).
17. Hayashi, S. & Yamamori, T. Forecast of time-to-slope failure by the a-tr method. *J. Jpn. Landslide Soc.* **28**, 1–8 (1991).
18. Tang, R. *et al.* The failure mechanism of the Baishi landslide in Beichuan County, Sichuan, China. *Sci. Rep.* **14**, 17482 (2024).
19. Li, B. *et al.* Mechanism of mining-induced landslides in the karst mountains of Southwestern China: a case study of the Baiyan landslide in Guizhou. *Landslides* **20**, 1481–1495 (2023).
20. Loew, S. *et al.* Early warning and dynamics of compound rockslides: lessons learnt from the Brienz/Brinzauls 2023 rockslope failure. *Landslides* (2024) doi:10.1007/s10346-024-02380-z.
21. Carlà, T. *et al.* Perspectives on the prediction of catastrophic slope failures from satellite InSAR. *Sci. Rep.* **9**, 14137 (2019).
22. Saito, M. Forecasting the time of occurrence of a slope failure. in *Proceedings of the 6th International Conference of Soil Mechanics and Foundation Engineering* 537–541 (1965, Montreal, 1965).
23. Carlà, T. *et al.* Rockfall forecasting and risk management along a major transportation corridor in the Alps through ground-based radar interferometry. *Landslides* **16**, 1425–1435 (2019).
24. Cicoira, A. *et al.* In situ observations of the Swiss periglacial environment using GNSS instruments. *Earth Syst. Sci. Data* **14**, 5061–5091 (2022).
25. Brawner, C. O. & Stacey, P. F. Hogarth Pit Slope Failure, Ontario, Canada. in *Developments in Geotechnical Engineering* (ed. Voight, B.) vol. 14 691–707 (Elsevier, 1979).
26. Ryan, T. M. & Call, R. D. Applications of rock mass monitoring for stability assessment of pit slope failure. in *Proceedings of the 33rd U.S. Symposium on Rock Mechanics* (OnePetro, 1992).
27. Chen, M., Huang, D. & Jiang, Q. Slope movement classification and new insights into failure prediction based on landslide deformation evolution. *Int. J. Rock Mech. Min. Sci.* **141**, 104733 (2021).
28. Yamaguchi, U. & Shimotani, T. A case study of slope failure in a limestone quarry. *Int. J. Rock Mech. Min. Sci. Geomech. Abstr.* **23**, 95–104 (1986).

29. Manconi, A. & Giordan, D. Landslide failure forecast in near-real-time. *Geomat. Nat. Hazards Risk* **7**, 639–648 (2016).
30. Kayesa, G. Prediction of slope failure at Letlhakane Mine with the Geomos slope monitoring system. in *Proceedings of the International Symposium on Stability of Rock Slopes in Open Pit Mining and Civil Engineering* (The South African Institute of Mining and Metallurgy, South African, 2006).
31. Fan, X. *et al.* Successful early warning and emergency response of a disastrous rockslide in Guizhou province, China. *Landslides* **16**, 2445–2457 (2019).
32. Intrieri, E. *et al.* The Maoxian landslide as seen from space: detecting precursors of failure with Sentinel-1 data. *Landslides* **15**, 123–133 (2018).
33. Gigli, G., Fanti, R., Canuti, P. & Casagli, N. Integration of advanced monitoring and numerical modeling techniques for the complete risk scenario analysis of rockslides: The case of Mt. Beni (Florence, Italy). *Eng. Geol.* **120**, 48–59 (2011).
34. Jacquemart, M. & Tiampo, K. Leveraging time series analysis of radar coherence and normalized difference vegetation index ratios to characterize pre-failure activity of the Mud Creek landslide, California. *Nat. Hazards Earth Syst. Sci.* **21**, 629–642 (2021).
35. Brown, I., Hittinger, M. & Goodman, R. Finite element study of the Nevis Bluff (New Zealand) rock slope failure. *Rock Mech.* **12**, 231–245 (1980).
36. Carey, J. The Progressive Development and Post-failure Behaviour of Deep-seated Landslide Complexes. (Durham University, 2011).
37. Zvelebill, J. & Moser, M. Monitoring based time-prediction of rock falls: Three case-histories. *Phys. Chem. Earth Part B Hydrol. Oceans Atmosphere* **26**, 159–167 (2001).
38. Carlà, T., Farina, P., Intrieri, E., Botsialas, K. & Casagli, N. On the monitoring and early-warning of brittle slope failures in hard rock masses: Examples from an open-pit mine. *Eng. Geol.* **228**, 71–81 (2017).
39. Fujisawa, K., Marcato, G., Nomura, Y. & Pasuto, A. Management of a typhoon-induced landslide in Otomura (Japan). *Geomorphology* **124**, 150–156 (2010).
40. Loew, S., Gschwind, S., Gischig, V., Keller-Signer, A. & Valenti, G. Monitoring and early warning of the 2012 Preonzo catastrophic rockslope failure. *Landslides* **14**, 141–154 (2017).
41. Royán, M. J., Abellán, A. & Vilaplana, J. M. Progressive failure leading to the 3 December 2013 rockfall at Puigcercós scarp (Catalonia, Spain). *Landslides* **12**, 585–595 (2015).

42. Mazzanti, P., Bozzano, F., Cipriani, I. & Prestininzi, A. New insights into the temporal prediction of landslides by a terrestrial SAR interferometry monitoring case study. *Landslides* **12**, 55–68 (2015).
43. Okamoto, T. *et al.* Displacement properties of landslide masses at the initiation of failure in quick clay deposits and the effects of meteorological and hydrological factors. *Eng. Geol.* **72**, 233–251 (2004).
44. Saito, M. *Evidencial Study on Forecasting Occurence of Slope Failure*. 1–23 (1979).
45. Nonveiller, E. The Vajont reservoir slope failure. *Eng. Geol.* **24**, 493–512 (1987).
46. Kristensen, L. *et al.* Movements, failure and climatic control of the Veslemannen rockslide, Western Norway. *Landslides* **18**, 1963–1980 (2021).
47. Kwan, D. Observations of the failure of a vertical cut in clay at Welland, Ontario. *Can. Geotech. J.* **8**, 283–298 (1971).
48. Xue, L. *et al.* New quantitative displacement criteria for slope deformation process: From the onset of the accelerating creep to brittle rupture and final failure. *Eng. Geol.* **182**, 79–87 (2014).
49. Kuo, H.-L. *et al.* Displacement evolution of failure and non-failure sliding rock slopes. *Landslides* **22**, 1213–1226 (2025).
50. Shen, B., King, A. & Guo, H. Displacement, stress and seismicity in roadway roofs during mining-induced failure. *Int. J. Rock Mech. Min. Sci.* **45**, 672–688 (2008).
51. Ouillon, G. & Sornette, D. The concept of 'critical earthquakes' applied to mine rockbursts with time-to-failure analysis. *Geophys. J. Int.* **143**, 454–468 (2000).
52. Malan, D. F., Napier, J. A. L. & Janse van Rensburg, A. L. Stope deformation measurements as a diagnostic measure of rock behaviour: A decade of research. *J. South. Afr. Inst. Min. Metall.* **107**, 743–765 (2007).
53. Walker, C. C., Becker, M. K. & Fricker, H. A. A high resolution, three-dimensional view of the D-28 Calving Event from Amery Ice Shelf with ICESat-2 and satellite imagery. *Geophys. Res. Lett.* **48**, e2020GL091200 (2021).
54. Pralong, A., Birrer, C., Stahel, W. A. & Funk, M. On the predictability of ice avalanches. *Nonlinear Process. Geophys.* **12**, 849–861 (2005).
55. Chmiel, M. *et al.* Seismic constraints on damage growth within an unstable hanging glacier. *Geophys. Res. Lett.* **50**, e2022GL102007 (2023).
56. Faillettaz, J., Funk, M. & Vagliasindi, M. Time forecast of a break-off event from a hanging glacier. *The Cryosphere* **10**, 1191–1200 (2016).

57. Dematteis, N., Giordan, D., Troilo, F., Wrzesniak, A. & Godone, D. Ten-year monitoring of the Grandes Jorasses glaciers kinematics. Limits, potentialities, and possible applications of different monitoring systems. *Remote Sens.* **13**, 3005 (2021).
58. Giordan, D., Dematteis, N., Allasia, P. & Motta, E. Classification and kinematics of the Planpincieux Glacier break-offs using photographic time-lapse analysis. *J. Glaciol.* **66**, 188–202 (2020).
59. Scambos, T. *et al.* Calving and ice-shelf break-up processes investigated by proxy: Antarctic tabular iceberg evolution during northward drift. *J. Glaciol.* **54**, 579–591 (2008).
60. Faillettaz, J., Pralong, A., Funk, M. & Deichmann, N. Evidence of log-periodic oscillations and increasing icequake activity during the breaking-off of large ice masses. *J Glaciol* **54**, 725–737 (2008).
61. Faillettaz, J., Funk, M. & Sornette, D. Icequakes coupled with surface displacements for predicting glacier break-off. *J. Glaciol.* **57**, 453–460 (2011).
62. Meier, L., Jacquemart, M., Steinacher, R., Jäger, D. & Funk, M. Monitoring of the Weissmies glacier before the failure event of September 10, 2017 with Radar Interferometry and high-resolution deformation camera. in *Proceedings of the International Snow Science Workshop* (Innsbruck, Austria, 2018).
63. Chadwick, W. W. *et al.* Geodetic monitoring at Axial Seamount since its 2015 eruption reveals a waning magma supply and tightly linked rates of deformation and seismicity. *Geochem. Geophys. Geosystems* **23**, e2021GC010153 (2022).
64. Cannata, A. *et al.* Pressurization and depressurization phases inside the plumbing system of Mount Etna volcano: Evidence from a multiparametric approach. *J. Geophys. Res. Solid Earth* **120**, 5965–5982 (2015).
65. Carracedo, J. C. *et al.* The 2011–2012 submarine eruption off El Hierro, Canary Islands: New lessons in oceanic island growth and volcanic crisis management. *Earth-Sci. Rev.* **150**, 168–200 (2015).
66. Sudo, Y. *et al.* Seismic activity and ground deformation associated with 1995 phreatic eruption of Kuju Volcano, Kyushu, Japan. *J. Volcanol. Geotherm. Res.* **81**, 245–267 (1998).
67. Bell, A. F., Greenhough, J., Heap, M. J. & Main, I. G. Challenges for forecasting based on accelerating rates of earthquakes at volcanoes and laboratory analogues. *Geophys. J. Int.* **185**, 718–723 (2011).

68. Ratdomopurbo, A. *et al.* Overview of the 2006 eruption of Mt. Merapi. *J. Volcanol. Geotherm. Res.* **261**, 87–97 (2013).
69. Surono *et al.* The 2010 explosive eruption of Java's Merapi volcano—A '100-year' event. *J. Volcanol. Geotherm. Res.* **241–242**, 121–135 (2012).
70. Miller, T. P. & Chouet, B. A. The 1989–1990 eruptions of Redoubt Volcano: An introduction. *J. Volcanol. Geotherm. Res.* **62**, 1–10 (1994).
71. Bull, K. F. & Buurman, H. An overview of the 2009 eruption of Redoubt Volcano, Alaska. *J. Volcanol. Geotherm. Res.* **259**, 2–15 (2013).
72. Geist, D. J. *et al.* The 2005 eruption of Sierra Negra volcano, Galápagos, Ecuador. *Bull. Volcanol.* **70**, 655–673 (2008).
73. Bell, A. F. *et al.* Caldera resurgence during the 2018 eruption of Sierra Negra volcano, Galápagos Islands. *Nat. Commun.* **12**, 1397 (2021).
74. Swanson, D. A. *et al.* Predicting eruptions at Mount St. Helens, June 1980 through December 1982. *Science* **221**, 1369–1376 (1983).
75. Bell, A. F., Naylor, M. & Main, I. G. The limits of predictability of volcanic eruptions from accelerating rates of earthquakes. *Geophys. J. Int.* **194**, 1541–1553 (2013).
76. Young, S. R. *et al.* Overview of the eruption of Soufriere Hills Volcano, Montserrat, 18 July 1995 to December 1997. *Geophys. Res. Lett.* **25**, 3389–3392 (1998).
77. Nakada, S., Shimizu, H. & Ohta, K. Overview of the 1990–1995 eruption at Unzen Volcano. *J. Volcanol. Geotherm. Res.* **89**, 1–22 (1999).
